# UM171 cooperates with PIM1 inhibitors to restrict HSC expansion markers and suppress leukemia progression

**DOI:** 10.1038/s41420-022-01244-6

**Published:** 2022-11-05

**Authors:** Anling Hu, Jian Gao, Krishnapriya M. Varier, Babu Gajendran, Fei Jiang, Wuling Liu, Chunlin Wang, Xiao Xiao, Yanmei Li, Eldad Zacksenhaus, Sajjad Ali, Yaacov Ben-David

**Affiliations:** 1 State Key Laboratory for Functions and Applications of Medicinal Plants, Guizhou Medical University, Guiyang, 550014 Guizhou Province People’s Republic of China; 2 The Key Laboratory of Chemistry for Natural Products of Guizhou Province and Chinese Academic of Sciences, Guiyang, 550014 Guizhou Province People’s Republic of China; 3 School of Pharmaceutical Sciences, Guizhou Medical University, Guiyang, 550025 Guizhou Province People’s Republic of China; 4 Department of Medicine, University of Toronto/Division of Advanced Diagnostics, Toronto General Research Institute—University Health Network, Toronto, Ontario Canada; 5 Department of Chemistry, Karakoram International University, Gilgit, 15100 Pakistan

**Keywords:** Targeted therapies, Acute myeloid leukaemia

## Abstract

The pyrimido-indole derivative UM171 promotes human Hematopoietic Stem Cells Expansion (HSCE), but its impact on leukemia is not known. Herein, we show in a mouse model of erythroleukemia that UM171 strongly suppresses leukemia progression. UM171 inhibits cell cycle progression and apoptosis of leukemic cells in culture. The effect of UM171 on leukemia differentiation was accompanied by increased expression of HSCE markers. RNAseq analysis combined with Q-RT-PCR and western blotting revealed that the PIM1 protein kinase is highly elevated in response to UM171 treatment. Moreover, docking analysis combined with immunoprecipitation assays revealed high binding affinity of UM171 to PIM1. Interestingly, pan-PIM kinase inhibitors counteracted the effect of UM171 on HSCE marker expression and PIM1 transcription, but not its suppression of leukemic cell growth. Moreover, combination treatment with UM171 and a pan-PIM inhibitor further suppressed leukemic cell proliferation compared to each drug alone. To uncover the mechanism of growth inhibition, we showed strong upregulation of the cyclin-dependent kinase inhibitor P21^CIP1^ and the transcription factor KLF2 by UM171. In accordance, KLF2 knockdown attenuated growth inhibition by UM171. KLF2 upregulation by UM171 is also responsible for the activation of P21^CIP1^ in leukemic cells leading to a G1/S arrest and suppression of leukemogenesis. Thus, suppression of leukemic growth by UM171 through KLF2 and P21^CIP1^ is thwarted by PIM-mediated expansion of leukemic stemness, uncovering a novel therapeutic modality involving combined UM171 plus PIM inhibitors.

## Introduction

Umbilical Cord blood (CB) transplant is used to treat numerous hematological malignancies [1]. In these malignancies, CB transplant eliminates the need for HLA matching and decreases the risk of chronic Graft-Versus-Host Disease (GVHD) [2]. Recent studies have identified the long-term-repopulating hematopoietic stem cells (LT-HSCs) in CB capable of generating all mature blood cells [3]. In the past decade, conditions were established to grow and expand CB in culture and to demonstrate their long-term engraftment capacity in vivo [4–7].

Recently, cultured CB cells were used in a drug screening assay to identify small molecule/compounds capable of expanding LT-HSC–stimulating activities. One such, compound is a pyrimido-indole derivative, UM171, which acts as a self-renewal agonist to expand LT-HSCs and enhance multilineage blood cell reconstitution in mice [7]. A similar HSC cell renewal and reconstitution ability by UM171 has also recently been demonstrated in humans [8]. While the mechanism by which UM171 expands LT-HSC is not yet known, a small subset of human cord blood CD34^+^ cells express endothelial protein C receptor (EPCR/CD201/PROCR) when exposed to UM171. EPCR is thus a cell culture-compatible marker of UM171-expanded human cord blood HSCs [9]. Another study demonstrated that UM171 expands distinct types of myeloid and NK progenitors from human pluripotent stem cells [10]. Knockdown of the Lysine-Specific histone demethylase 1 A (*LSD1/KDM1*) in mice results in the expansion of HSCs similar to the phenotype seen in UM171-treated mice [11], suggesting it is a possible target of UM171.

Herein, we report the identification of UM171 as a potent anti-neoplastic growth agent against diverse types of hematological tumor cell lines. Although UM171 induces expression of HSC expansion markers, it also displays anti-neoplastic activity. Expression analysis revealed that UM171 induces not only classical HSC gene markers, but also the PIM1 proto-oncogene in leukemic cells. PIM1 was first identified as a site of proviral integration for the Moloney Murine Leukemia virus [12]. Later, other variants of the *PIM* (*PIM2-PIM3*) are overexpressed in various cancers [13]. *PIM* genes are serine/threonine kinases that promote growth and survival in multiple cell types, and are implicated in the pathogenesis of multiple diseases [14]. In addition, PIM1-PIM3 have been implicated in HSC self-renewal as knock-down of all three genes in mice disrupts HSC expansion and self-renewal [15]. Conversely, PIM1 overexpression in mice leads to cancer development by inhibiting apoptosis, promoting cell proliferation, and inducing of genomic instability [16]. PIMs is therefore a potential therapeutic target for the treatment of cancers in which it is induced; several PIM kinase inhibitors have been identified, including the pan-PIM inhibitors AZD1208, SC1776, LGH447 and TP3654, some of which are currently assessed in clinical trials [13].

In this study, we demonstrate high-affinity binding of UM171 to PIM1. This interaction activates PIM1, leading to elevated expression of HSC markers, which in turn can be blocked by pan-PIM inhibitors. In an animal model of leukemia, UM171 exhibits strong anti-leukemia activity that can be further enhanced upon the combination with pan-PIM inhibition. The antileukemic activity of UM171 was linked to increased expression of genes associated with cell cycle inhibition and induction of apoptosis. We propose that UM171 exerts its effect through two independent pathways (i) HSC expansion markers via PIM1, which could increase leukemia stemness; (ii) growth suppression via as yet to be defined target that controls the tumor suppressor genes KLF2 and P21^CIP1^. Combined UM171 plus PIM-inhibitor therapy offers a new strategy to limit leukemic growth.

## Results

### Identification of UM171 as a strong anti-leukemia inhibitor of leukemic cell growth in culture and in a mouse model in vivo

**Fig. 1:**
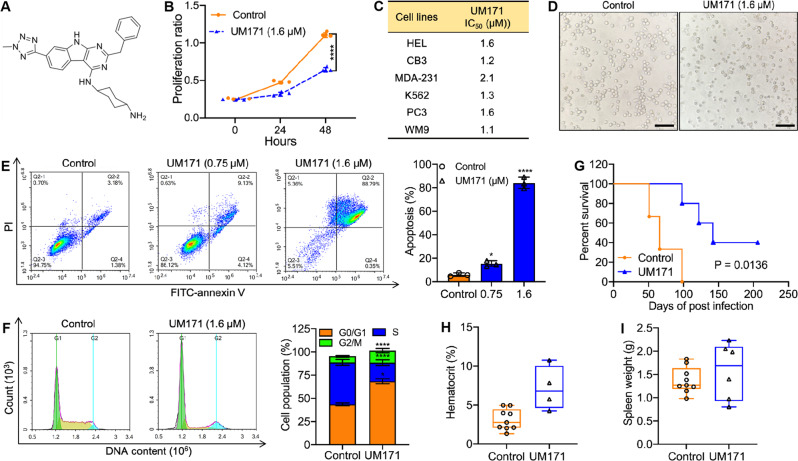
Inhibition of leukemogenesis by UM171 in culture and in vivo. **A** Structure of UM171. **B** UM171 at indicated concentration inhibits proliferation of HEL cells in culture. **C** IC_50_ analysis of UM171 for the indicated cell lines. **D** Macroscopic image of apoptotic cells after treatment of HEL cells with 1.6 μM of UM171 (original magnification X20, scale bars, 100 μm). **E** UM171 induces apoptosis of HEL cells at the indicated concentrations. **F** UM171 induces G_1_/S cell cycle arrest of HEL cell at indicated concentrations. **G** Newborn BALB/c mice infected with F-MuLV were treated for two weeks with UM171 (3 mg/ Kg), 5 weeks postviral infection, and plotted after all mice succumbed to leukemia. **H**, **I** UM171 treated mice with their hematocrit values (**H**) and tumor volume (**I**) at the time of death.

UM171 (structure shown in Fig. 1A) was originally recognized for its Hematopoietic Stem Cell Expansion (HSCE) capacity, yet its effect on cell proliferation and survival is unknown. Remarkably, we found that UM171 significantly inhibited the proliferation of the leukemia cell line, HEL, in culture (Fig. 1B). IC_50_ analysis of UM171 for erythroleukemia (HEL, CB3, K562), breast (MDA-231), prostate (PC3), and melanoma (WM9) cell lines demonstrated diverse anti-cancer activity (Fig. 1C). UM171 induced dose-dependent apoptosis 48 hours post-drug treatment (Fig. 1D-E) and G1 cell cycle arrest of HEL cells in culture as revealed by AnnexinV staining and PI flow cytometry, respectively (Fig. 1F).

In a mouse model of erythroleukemia induced by Friend Murine Leukemia Virus (F-MuLV), treatment with UM171 (3 mg/kg; 3x/week) significantly delayed erythroleukemia progression (*P* = 0.0136; Fig. 1G). In this experiment, UM171-treated mice that succumbed to the disease exhibited lower anemia with higher hematocrit (Fig. 1H) and relatively large spleen tumor mass (Fig. 1I). This is consistent with the fact that untreated F-MuLV-infected control mice develop severe anemia associated with lower hematocrit and large tumors in the spleen [17]. Indeed, spleen size similar to control at the disease endpoint suggests no general toxicity of the drug in vivo [17].

### UM171 induces expression of HSCs markers in treated leukemic cells

Cancer stem cells are known to resist conventional and targeted therapy [18]. We tested whether UM171 with its strong anti-leukemic activity was still capable of inducing stemness through upregulation of markers of HSCs in HEL cells. Remarkably, at different doses that inhibited leukemic cell proliferation in culture, UM171 treatment robustly induced HSC markers.

**Fig. 2:**
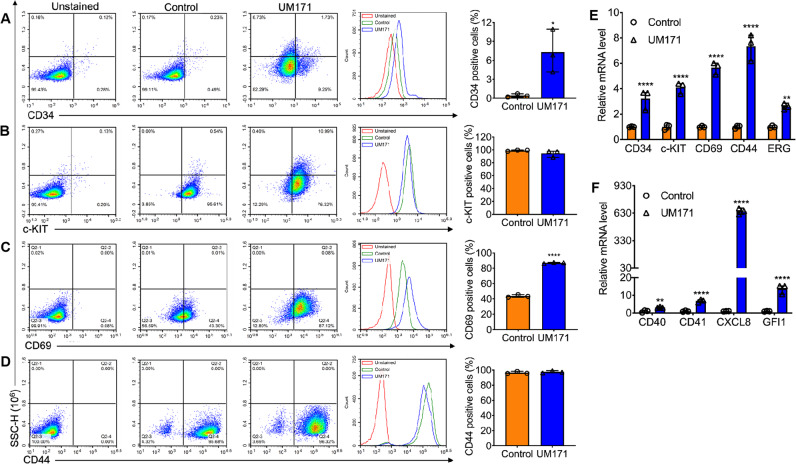
Induction of stem cell markers by UM171 in HEL cells. **A**–**D** The Bi-potential Megakaryocytic Erythroid Progenitor (MEP) derived HEL cell line was treated with UM171 (6 μM), DMSO for 24 hours and subjected to flow cytometry analysis for the stem cell markers CD34 (**A**), c-KIT (B), CD69 (**C**) and CD44 (**D**). **E**, **F** Q-RT-PCR analysis of HEL cell treated with UM171 (3 μM) and DMSO for expression of the stem cell markers CD34, c-KIT, CD69, CD44, ERG (**E**) and CD40, CD41, CXCL8 and GFI1 (**F**).

By flow cytometry, UM171 reduced expression of the erythroid differentiation genes CD71 and CD235 (Supplemental Fig. 1A, B). This may be the result of de-differentiation of HEL cells into earlier progenitors, as previously seen through overexpression of Fli-1 [19]. Such de-differentiation was further supported by the suppression of HBA1 (α1-globin), HBA2 (β-globin), HBQ1(theta-1 globin), and HBZ (Zeta globin) expression in the UM171-treated erythroleukemic cells (Supplemental Fig. 1C–F). UM171 also induced expression of CD34, CD69, c-KIT, and CD44, which are known markers of HSCs and early progenitors (Fig. 2A–D) [20–22]. These results were further confirmed using Q-RT-PCR for the CD34, CD69, c-KIT, and CD44 genes (Fig. 2E). Q-RT-PCR analysis also confirmed induction by UM171 of other HSC markers including CD40, CD41, CXCL8 (IL8), GFI1, and ERG (Fig. 2F). In contrast to ERG, FLI1 expression was not affected by UM171 (data not shown). These results establish UM171 as a potent leukemic de-differentiation and HSC marker expansion agent.

### UM171 specifically binds to PIM1 to induce its expression and increase its stem cell expansion activity

**Fig. 3:**
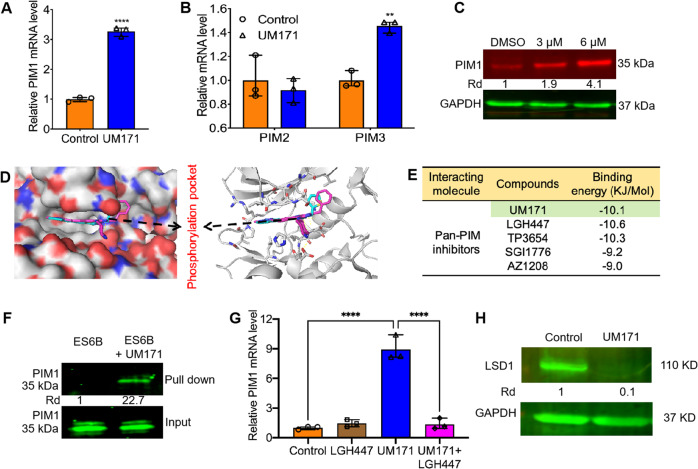
UM171 binds to PIM1 and activates its phosphorylation. **A** Q-RT-PCR analysis of HEL cells treated with UM171 (6 μM) for expression of the *PIM1*. **B** Q-RT-PCR analysis of HEL cells treated with UM171 (6 μM) for expression of the *PIM2*, and *PIM3* genes. **C** Western blot of HEL cells treated with the indicated concentration of UM171 compound. GAPDH was used as a loading control. **D** Molecular docking of the compound UM171 and PIM1 (PDB:5O12). The phosphorylation site of PIM1 kinase was shown as insert. **E** The molecular binding energy between PIM1 and UM171 as well as pan-PIM inhibitors LGH447, TP3654, SGI1776 ad AZ1208. **F** Binding of PIM1 to UM171 in pull-down experiment using affinity ES6B beads. **G** Q-RT-PCR analysis of HEL cells treated with UM171 (6 uM), LGH447(5 μM), and UM171 + LGH447 for 24 hours for expression of PIM1. **H** Western blot of HEL cells treated with UM171 and DMSO for expression of LSD1.

To uncover the underlying mechanism of stem cell expansion and leukemia inhibition by UM171, HEL cells were treated with this compound (1.6 µM) for 24 hours and subjected to RNAseq analysis. This analysis identified over 600 differentially expressed genes (DEGs) (Supplemental table 3). The proto-oncogene PIM1 was induced about 3-folds. Dose-dependent analysis confirmed that UM171 induced PIM1 protein and mRNA expression in HEL cells (Fig. 3A). UM171 also slightly induced the PIM-related gene *PIM3* but had no discernibly affect on *PIM2* (Fig. 3B). Consistent with this, UM171 induced PIM1 protein expression in a dose-dependent manner (Fig. 3C).

To determine if PIM1 is a direct target of UM171, a docking analysis was conducted, and a strong affinity of UM171 to the phosphorylation pocket of PIM1 was observed (Fig. 3D), with the binding energy of −10.1 KJ/mol (Fig. 3E). As, PIM1 is a phospho-protein and inhibitors of this kinase are available, we also compared the binding affinity of these inhibitors to the PIM1 kinase pocket (Fig. 3E). Although, all 4 inhibitors (LGH447, TP3654, SG11776, AZ1208) have a strong affinity to the PIM1 kinase domain, their affinity was lower than that of UM171 (Fig. 3E). In a pull-down assay, the compound was bound to ESB6 beads (UM171-ESB6), as previously described [23]. UM171-ESB6 beads were incubated with leukemia extracts and subjected to immunoprecipitation. Western blot detected a band corresponding to PIM1 bound to the beads. As control, ESB6 beads treated with the same cell extracts gave rise to no band (Fig. 3F). A recent study has demonstrated that PIM1 induces STAT3, raising the possibility that UM171 may also regulate the latter [24]. However, UM171 slightly reduced the expression of STAT3 and its phosphorylation (Supplemental Fig. 2). Interestingly, while UM171 strongly induced PIM1 transcription in HEL cells, this induction was completely inhibited when cells were co-treated with UM171 and the pan-PIM inhibitor LGH447 (Fig. 3G). This result suggests that the binding of UM171 to PIM1 causes activation of this kinase and auto-regulation of its own transcription.

Previously, endothelial protein C receptor (EPCR/CD201/PROCR) expression in CD34^+^ stem cells was identified as a target of UM171, and as possible mediator of HSC expansion [9]. Indeed, EPCR expression was significantly induced by UM171 and its expression was suppressed when HEL cells were co-incubated with the pan-PIM inhibitor LGH447, indicating that this pathway is likely to be downstream of PIM1 (supplemental Fig. 3A). Another study has recently demonstrated that the lysine-specific demethylase 1 A (LSD1/RCOR1) restricts ex vivo propagation of HSCs, suggesting this gene as another target of UM171 [11]. Another recent study revealed that UM171 potentiates the activity of a CULLIN3-E3 ubiquitin ligase (CRL3) complex to degrade Kelch/BTB domain protein KBTBD4. CULLIN3-KBTBD4 ubiquitin ligase activated by UM171 then specifically targets LSD1 corepressor complex for proteasomal degradation, causing HSC expansion [25]. We found that while LSD1 transcription was not induced by UM171, the LSD1-specific inhibitor (GSK-LSD1) was also unable to change the expression of this gene with or without UM171 in leukemic cells (Supplemental Fig. 3B). The pan-PIM inhibitor LGH447 also did not change the level of LSD1 mRNA in UM171 treated cells (Supplemental Fig. 3C). However, at the protein level, UM171 strongly inhibited LSD1 expression, and this was not affected by GSK-LSD1 or LGH447 treatment (Fig. 3H and supplemental Fig. 4). These results suggest that while EPCR is downstream of PIM1, LSD1 likely employs an alternative pathway to expand HSCs in response to UM171.

### Pan-PIM inhibitors suppress HSC and de-differentiation markers induced by UM171

**Fig. 4:**
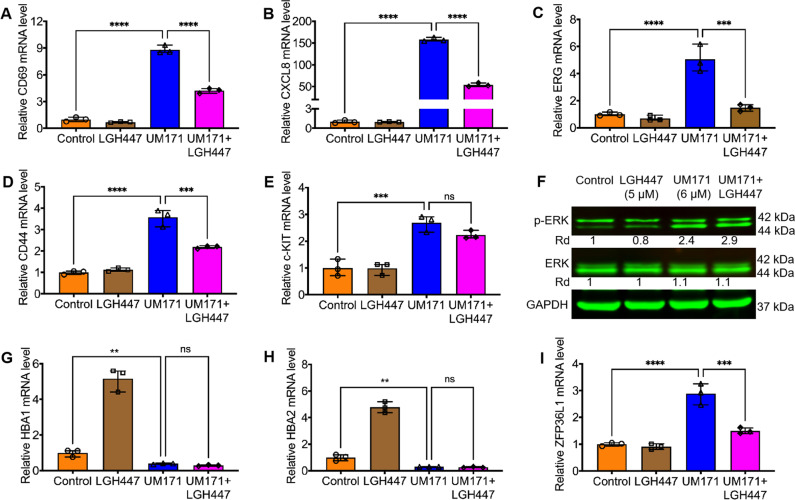
UM171 induces markers of HSCs in HEL cells. **A**–**E** HEL cells were treated with UM171 (6 μM), LGH447(5 μM), and UM171 + LGH447 for 24 hours and subjected to Q-RT-PCR analysis for the indicated genes. **F** HEL cells were treated with UM171 (6 μM), LGH447(5 μM), and UM171 + LGH447 for 24 hours and subjected to western blot analysis for detection of ERK and Phospho-ERK. GAPDH was used as a loading control. **G**–**I** Q-RT-PCR analysis of HEL cells treated with the indicated compounds for expression of HBA1 (**G**), HBA2 (**H**), ZFP36L1 (**I**).

The abovementioned results suggest that UM171 activates PIM1, leading to de-differentiation and expansion of HSCs. To further corroborate this observation, we asked whether PIM1 inhibition by LGH447 could reverse the effect of UM171 on HSC marker expression. HEL cells treated for 24 hours with both compounds exhibited significantly reduced expression of CD69, CXCL8, ERG, CD44, relative to UM171-treated cells alone (Fig. 4A–D). c-KIT expression while induced by UM171 in both Q-RT-PCR (Fig. 4E) and western blot (Supplemental Fig. 5), UM171 + LGH447 causing lower expression, but not significant (Fig. 4E and supplemental Fig. 5). However, GSK-LSD1 diminished some of the UM171-mediated induction of CXCL8 and CD44, but not c-KIT and CD69 (Supplemental Fig. 6). These results further support a role for PIM1 as a critical regulator of HSC marker expansion and suggest an independent and overlapping mechanism of HSCE by LSD1.

Previously, ERK/MAPK was identified as a target of PIM1 activation [26]. UM171 treatment induced phospho-ERK expression, and this induction was partially reversed by LGH447 (Fig. 4F). LGH447 alone slightly reduced phospho-ERK level (Fig. 4F). UM171 treatment strongly inhibited the expression of erythroid differentiation genes HBA1 and HBA2 (Fig. 4G, H) that was not restored when cells were treated with a combination of UM171 plus LGH447. In this experiment, LGH447 was significantly induced HBA1 and HBA2 expression. These results indicate that the suppression of erythroid differentiation by UM171 is independent of PIM1. ZFP36L1, a member of ZFP36 zinc finger protein family, was previously shown to negatively regulate erythroid differentiation of CD34^+^ hematopoietic stem cells by interfering with the Stat5b pathway [27]. To our surprise, UM171 treatment caused a drastic upregulation of ZFP36L1 expression that was restored upon treatment with LGH447 (Fig. 4I). These results suggest that α-globin genes (HBA1 and HBA2) regulation by ZFP36L1 may be mediated through PIM1 activation by UM171. Our results also demonstrate that growth inhibition by UM171 is a regulated process, not a nonspecific cell toxicity as originally proposed [7].

### UM171 induces the cell cycle inhibitor P21^CIP1^ independently of PIM1 activation

**Fig. 5:**
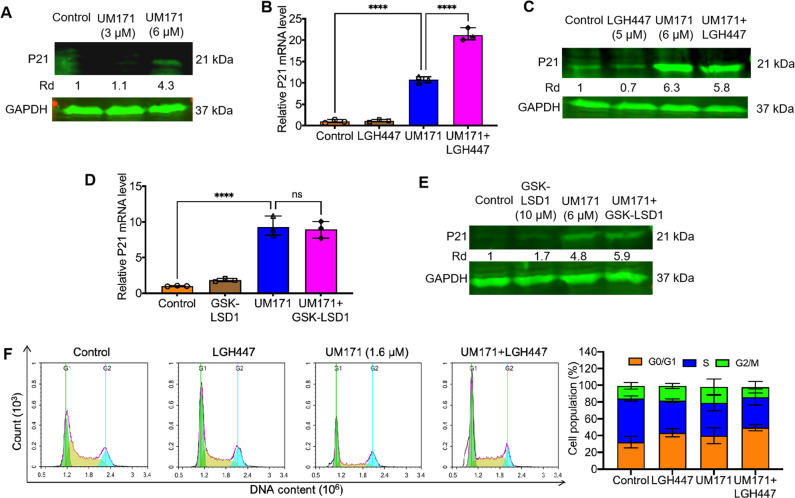
UM171 upregulates expression of the cell cycle inhibitor p21^CIP1^ independent of PIM1 activation. **A** HEL cells were treated with indicated doses of UM171 and subjected to western blot for P21^CIP1^ and PIM1 expression. **B**, **C** HEL cells were treated with UM171, LGH447, and UM171 + LGH447 for 24 hours and subjected to Q-RT-PCR (B) or western blot (**C**) analysis for the indicated genes. **D**, **E** HEL cells were treated with UM171, GSK-LDS1 (10 μM), and P21^CIP1^ levels were determined by Q-RT-PCR (**D**) and western blotting (**E**). **F** Cell cycle arrest of HEL cells treated with UM171, LGH447 and UM171 + LGH447. Right panel shows the percentage of cells in different phases of the cell cycle.

The cyclin-dependent kinase 1 A inhibitor P21^CIP1^/CDKN1A was previously reported to be suppressed by PIM1 [28, 29]. However, UM171 treatment of HEL cells resulted in induction of both PIM1 and P21^CIP1^ expression in a dose-dependent manner (Fig. 5A). This result is consistent with the G_1_/S cell cycle arrest induced by UM171 (Fig. 1E). While treatment with LGH447 alone reduced P21^CIP^, UM171 plus LGH447 revealed no effect on P21^CIP1^ protein level, but a slightly increased transcription of this cell cycle inhibitor (Fig. 5B,C). UM171-induced P21^CIP1^ expression was also remained unchanged in cells co-treated with GSK-LSD1 inhibitor at both the transcription and protein level (Fig. 5D–E). Treatment with combination of UM171 and LGH447 caused a G_1_/S arrest similar to UM171-treated cells (Fig. 5F). LGH447 also induced a G_1_/S arrest (Fig. 5F) that was independent of P21^CIP1^ upregulation (Fig. 5C). These results suggest that in addition to PIM1, UM171 has other target(s) that induce P21^CIP1^ expression and cell cycle arrest.

### UM171 induces expression of KLF2 that inhibits leukemic cell proliferation

**Fig. 6:**
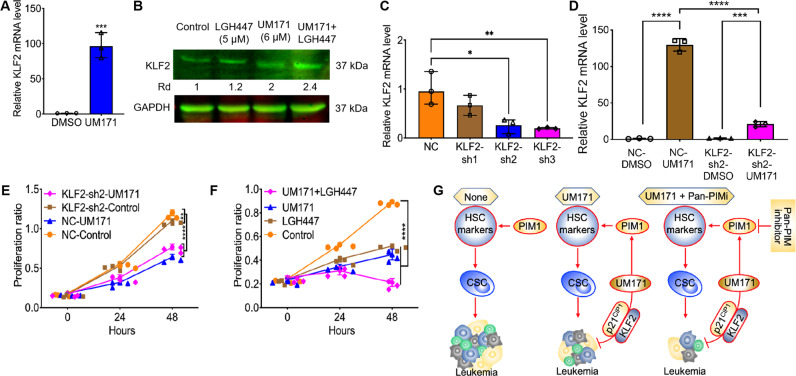
Induction of KLF2 by UM171 inhibit cell proliferation in culture. **A** Treatment of HEL cells with UM171 (6 μM) induces KLF2 expression, as determined by Q-RT-PCR. **B** KLF2 induction was partially blocked by treatment with LGH447 (5 μM), as determined by western blot. **C** Inhibition of KLF2 expression in HEL cells using shRNA constructs (KLF2-sh1, KLF2-sh2, KLF2-sh3), as determined by Q-RT-PCR. **D** Induction of KLF2 by UM171 was moderated in KLF2-sh2 cells, determined by Q-RT-PCR. **E** KLF2 knockdown moderated suppression of KLF2-sh2 cell proliferation by UM171 when compared to the control scrambled cells. **F** Growth suppression by UM171 was further accelerated when co-treated with LGH447(5 μM) in the culture of HEL cells. **G** Depicted diagram of growth suppression by UM171. UM171 through binding to PIM1 activates HSCE markers and cancer stemness by increasing cancer stem cells (CSCs) and via unknown target increases the expression of P21^cip1^ and KLF2, leading to growth suppression. Cancer stemness induction by UM171 may partially interfere with growth suppression by UM171 and blocking CSC by a pan-PIM inhibitor should further accelerate leukemia inhibition by the compound.

Our RNAseq analysis also identified strong upregulation of the Kruppel-like factor 2 (KLF2) transcription factor in UM171-treated HEL cells (Fig. 6A). KLF2 induction by UM171 was slightly increased upon co-treatment with LGH447 (Fig. 6B). To ask whether KLF2 induction by UM171 interferes with cell proliferation, this transcription factor was knocked downed in HEL cells using 3 lentivirus shRNAs, among which KLF2-sh2 and KLF2-sh3 variants showed strongest mRNA downregulation (Fig. 6C). After treatment with UM171, KLF2-sh2 cells exhibited lower KLF2 upregulation compared to scrambled control cells (Fig. 6D). Accordingly, the growth suppression induced by UM171 in scrambled control cells was significantly attenuated in KLF2-sh2 cells (Fig. 6E). These results revealed that growth suppression by UM171 was partially mediated through upregulation of KLF2 in leukemic cells. Similar to scrambled control cells, UM171 strongly upregulated the expression of c-KIT, CD69 and CD44 in KLF2-sh2, suggesting no role for KLF2 in HSCE gene regulation (Supplemental figure 7A-C). Moreover, KLF2 induction by UM171 was not moderated by treatment with GSK-LSD1 (Supplemental figure 8). Interestingly, the induction of globin HBA1 and HBA2 genes was strongly alleviated in KLF2-sh2 by UM171 (Supplemental figure 7D,E). This effect on globin expression was independent of FLI1, as its expression was previously shown to block erythroid differentiation (Supplemental figure 7F) [17, 30]. Similarly, induction of P21^CIP1^ by UM171 is reduced in KLF2-sh2 cells compared to scrambled control cells (Supplemental Figure 7G). This result is consistent with a previous observation demonstrating higher expression of P21^CIP1^ as the mechanism of growth suppression by KLF2 [31].

While UM171 strongly inhibited cell proliferation, the addition of LGH447 further increased this growth suppression in culture (Fig. 6F). In this experiment, LGH447 also inhibited HEL cell proliferation. These results reveals that a combination of UM171 plus pan-PIM inhibitors had a better therapeutic benefit in leukemic inhibition than either of these drugs alone. As depicted in Fig. 6G, in addition to activating stem cell expansion markers through PIM1 activation, UM171 induced growth arrest and inhibition of leukemogenesis through as yet unknown target(s). Hematopoietic expansion activity of UM171, which resembles cancer stemness [18], may positively affect leukemic cell proliferation. Inhibiting this leukemia stemness activity using pan-PIM inhibitors synergizes with UM171 to efficiently block leukemia progression.

## Discussion

UM171 was originally discovered as a HSCE compound to enhance stem cell engraftment in patients [7]. We herein show that in addition to HSCE function, UM171 also possesses strong anti-leukemia activity in culture and in a leukemia animal model. The HSCE activity found to be mediated partly through binding of UM171 to PIM1, leading to the activation of this kinase that can be blocked by pan-PIM inhibitors. This HSCE ability is independent of the anti-leukemia activity that is generated through an alternative pathway. This new anti-proliferative property of the compound triggers upregulation of cell cycle inhibitor P21^CIP1^ and transcription factor KLF2, partly involved in leukemia suppression. These studies for the first time revealed antileukemia activity of UM171 and identified PIM1 as a potential target of this compound during HSCE activity.

Previously, EPCR and LSD1 were identified as potential targets of UM171 for HSCE [9, 11]. Our study shows that EPCR is a downstream target of PIM1 also involved in HSCE. LSD1 inhibition by UM171, however, may induce HSCE through a PIM1-independent mechanism. This conclusion is consistent with the fact that the PIM1-PIM3 genes are associated with stem cells survival and differentiation. Knockdown of all the three *PIM (PIM1-3*) genes in mice cause a severe defect in HSC self-renewal and expansion [15]. UM171 clearly induces the expression of classical markers of HSCs in leukemia cells that are blocked by a pan-PIM inhibitor. This effect likely increases the tumor stemness, compromising the anti-leukemic activity of UM171. Treatment of erythroleukemia HEL cells with UM171 also causes de-differentiation associated with lower expression of erythroid-specific genes. Excitingly, de-differentiation by UM171 was not reversible by a pan-PIM inhibitor suggesting off-target activity resulting from changes in the expression of genes by the compound that interferes with the maturation process.

RNAseq analysis identified over 600 DEGs in leukemic cells treated with UM171 versus control. While many of these genes are likely to be associated with HSCE, others may be involved in leukemia inhibition property and other functions of UM171. Among these DEGs, the expression of cell cycle inhibitor P21^CIP1^ was significantly induced that was not blocked by a pan-PIM inhibitor. Higher P21^cip1^ expression was associated with G_1_/S cell cycle arrest that may in part responsible for leukemia inhibition by UM171. As P21^CIP1^ induces G_1_/S in a p53-dependent manner [32–35], HEL cells used in this study lost this tumor suppressor gene [36]. However, P21^CIP1^ is also known to induce G_1_/S arrest independent of p53 [37, 38]. As UM171 is also induced apoptosis, future studies may be needed to explore the underlying mechanism of cell death property of this compound.

UM171 also activated the expression of the growth suppressor *KLF2* gene in leukemic cells. KLF2 knockdown in leukemic cells can restrict growth inhibition by UM171, supporting a tumor suppressor function for this transcription factor. KLF2 induction is not blocked by UM171 and its upregulation is shown to play a role in leukemia inhibition. Again, to our surprise, induction of P21^CIP1^ by KLF2 was previously implicated in growth suppressor activity of this transcription factor [31, 39]. Similarly, KLF2 knockdown was shown here significantly inhibited P21^CIP1^ upregulation by UM171. These results then indicate KLF2 as a partial mediator of growth suppression by UM171. How UM171 induces KLF2 is an exciting area of research that needs to be investigated in future studies.

Low doses of UM171 inhibited leukemia cell proliferation in culture and suppressed leukemogenesis in vivo. Interestingly, previous studies implicated stemness as a hallmark of resistance to cancer therapy [40]. UM171 activation of HSCE markers that could elevate stemness then may restrict the leukemic inhibition capacity of UM171. Treatment of leukemic cells with the combination of UM171 and pan-PIM inhibitor significantly accelerated the anti-leukemic activity of UM171 when compared to each of these compounds alone (Fig. 6G). These results suggest that UM171 in the combination with a pan-PIM inhibitor would have a better therapeutic benefit than either of these compounds alone.

Taken together, this study showed that UM171 in addition to HSCE activity, possesses strong anti-leukemia ability in culture in an animal model. The HSCE activity was found to be mediated through the binding of UM171 to PIM1 kinase. While the receptor for anti-leukemic activity is still unknown, UM171 shown to activate the growth suppressor genes P21^CIP1^ and KLF2, which may partially be responsible for leukemia inhibition of this compound. As UM171 may activate stemness, the combination of UM171 and a pan-PIM inhibitor may have a better therapeutic benefit than either of these compounds individually. This study provides substantial insight into the mechanism of UM171 action in HSCE and anti-leukemia properties.

## Matarials and methods

### Cells culture and compound treatment

The human erythroleukemia HEL, K562, CB3, MDA-231, PC3, WM9, HEK293T cell lines were developed in house or previously obtained from ATCC (US), and maintained mycoplasma free. These cell lines cultured and maintained in Dulbecco’s Modified Eagle Medium supplemented with 5% fetal bovine serum (HyClone, GE Healthcare, US).

For drug therapy, HEL cells were treated with UM171 for the indicated times described in the text and a proliferation index was constructed via cell counting or MTT assay. The UM171 inhibitor was ordered from APE-BIO (A89505, US).

### Cell cycle and apoptosis analyses

HEL cells were seeded on 6 well plates and treated with the indicated compounds or vehicle control DMSO for 48 h. For cell cycle analysis, HEL cells were first fixed in cold 70% ethanol overnight at 4 °C. Fixed HEL cells were then incubated in PI staining buffer (TritonX-100, RNase A and PI) for 30 min at 37 °C, and immediately analyzed by flow cytometer (BD Biosciences, Franklin lakes, US). For apoptosis analysis, drug-treated HEL cells were stained with PI and Annexin V (BD FITC Annexin V Apoptosis Detection Kit I, #556547) for 15 minutes and analyzed by flow cytometer, as suggested by the product protocol.

### ShRNA construction and expression analysis

The shKLF2 lentivirus construction was conducted as previously described [30]. ShKLF2 and scrambled control plasmids were constructed by cloning the corresponding shRNA DNA fragment into the unique BcuI sites of the PLent-GFP expression vector (Vigene Bioscience, US). To produce shRNA lentiviruses, shRNA DNA (10 μg) was co-transfected with the packaging plasmids psPAX2 (5 μg) and pMD2.G (10 μg) (Addgene plasmid #12259 and #12260) into HEK293T cells, using Lipofectamine 2000 (11668-019, Thermo Fisher Scientific, US) [41]. Two days after transfection, the supernatant from confluent GFP-positive cells was collected and used to transduce HEL (1 × 10^6^) cells. The medium was changed 24 hours postinfection and transduced cells were selected in a medium containing puromycin (10 μg/ml) (Solarbio, cat.58-58-2, CN). The sequences for the shRNA lentiviruses are shown in supplementary table 1.

### Flow cytometry

For immunofluorescence staining, 1 × 10^6^ cells were stained with APC/FITC-conjugated antibodies for 40 min at RT, as previously described [41]. Immuno-stained cells were then washed twice with PBS and resuspended in 500 μl of phosphate-buffered saline and used for flow analysis. The following primary antibodies were used: human CD235-APC (Cat.551336), human CD34-APC (Cat.555824), human CD69-APC (Cat.555533), human CD44-APC (Cat.559942), human CD71-FITC (Cat.555536), human CD235a-APC (Cat.551336) were all purchased from BD Biosciences (BD Biosciences, NJ, US). c-KIT-APC (17-1171-82) was purchased from eBioscience (eBioscience Inc, CA, US). Flow cytometry was performed using a NovoCyte flow cytometer (ACEC Biosciences Inc, CA, US) and Novo-express software.

### RNA preparation and Q-RT-PCR

Total RNA was extracted from HEL cells using TRIzol reagent (Thermo Fisher Scientific, US). Total RNA was then used to synthesize cDNA in a reverse transcription reaction, using the PrimeScript RT reagent kit (Takara Bio, CN). Using these cDNAs, Q-RT-PCR was performed by employing the FastStart Universal SYBR-Green Master (Roche, CN) and the Step One Plus Real-time PCR system (Thermo Fisher Scientific, US) [30]. The expression of the genes was given as relative to *GAPDH*. The primer sequences used in the Q-RT-PCRs are listed in supplementary table 2. Three independent biological replicates were performed for all the Q-RT-PCRs, each in triplicate (*n* = 3).

### Western blot analysis

Western blotting was performed, as previously described [30]. The antibodies used were as follow: Polyclonal rabbit PIM1 (ab54503), FLI1 (ab133485) and ERK (ab184699) were purchased from Abcam (UK); the GAPDH (AB-P-R001) antibody was obtained from Goodhere Biotech (CN); c-KIT (18696-1-AP), LSD1 (20813-1-AP) and P21^CIP1^ (10355-1-AP) were obtained from Protein Technology (CN); p-ERK (9101 S), stat3 (4904), p-stat3 (9145), goat anti-mouse IgG (H + L) DyLight (TM) 680 (5470 s) and goat anti-rabbit IgG (H + L) DyLight (TM) 680 (5151 s) antibodies were obtained from Cell Signaling Technology (US). The dilution of antibodies was followed according to the manufacturer’s instructions. The Odyssey system (LI-COR Biosciences) was used to image and analyze the proteins on the western blot membranes.

### RNAseq analysis

RNAseq was carried out by using control cells and UM171-treated cells, as previously described with required modifications [41]. The data from this analysis is presented in supplemental table 3.

### leukemia drug therapy in mice

One day old BALB/c neonate mice were inoculated intraperitoneally (i.p.) with F-MuLV as previously described [17, 30]. Five weeks post viral infection, equal male and female mice (*N* = 6) were injected every other day for a total of seven inoculations with (3 mg/Kg body weight of UM171 and DMSO as control. Mice were then monitored for the development of severe leukemia. Mice showing signs of late-stage disease were sacrificed, spleens were removed and hematocrit was calculated, as previously described [30]. The percent survival, spleen weight and hematocrit values were calculated and presented in Fig. 1.

### Statistical analysis

The statistical analysis was conducted using a two-tailed Student t-test or a one-way ANOVA with Tukey’s post-hoc test, using Origin 3.5 software (Microcal Software). The threshold for significance was indicated within the figures as follow: *(*P* = < 0.05), **(*P* = < 0.001), ***(*P* = < 0.0001) and ****(*P* = < 0.00001). The results were expressed as mean ± the standard deviation from at least 3 independent experiments.

## Supplementary information

supplemental figure 1

supplemental figure 2

supplemental figure 3

supplemental figure 4

supplemental figure 5

supplemental figure 6

supplemental figure 7

supplemental figure 8

SUPPLEMENTAL FIGURES LEDENDS

Supplementary Table 1

Supplementary Table 2

supplementary table 3

full length of western blot

## Data Availability

All datasets presented in this study are included in the article/Supplementary Material.
