# Supplementary figures and images for: UM171 cooperates with PIM1 inhibitors to restrict HSC expansion markers and suppress leukemia progression
Source: Cell Death Discov. 2022 Nov 5;8:448. doi: 10.1038/s41420-022-01244-6 (PMC9637110; doi:10.1038/s41420-022-01244-6)

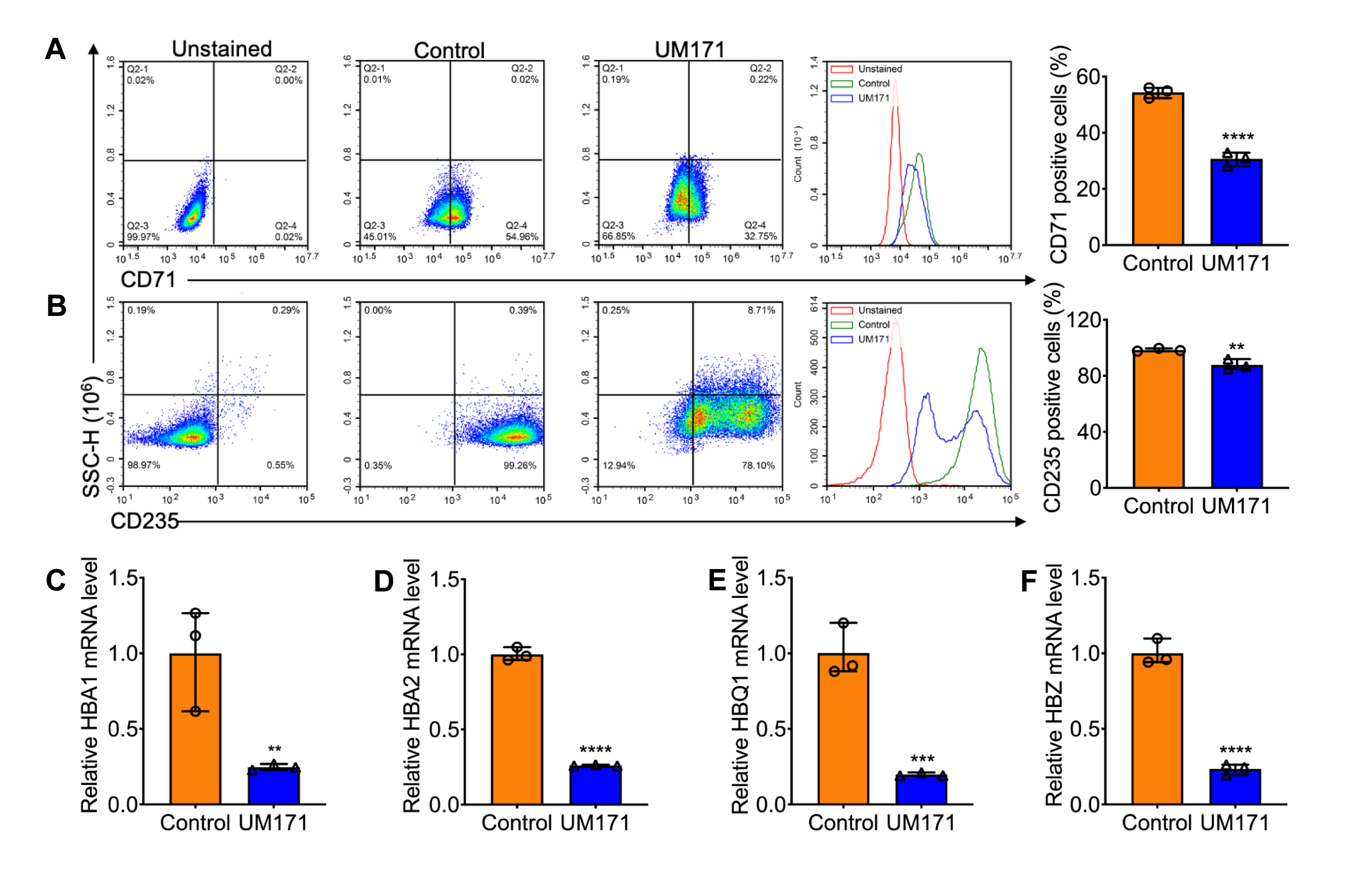

Supplement: Supplementary file 1 — supplemental figure 1 [file 41420_2022_1244_MOESM1_ESM.tif]

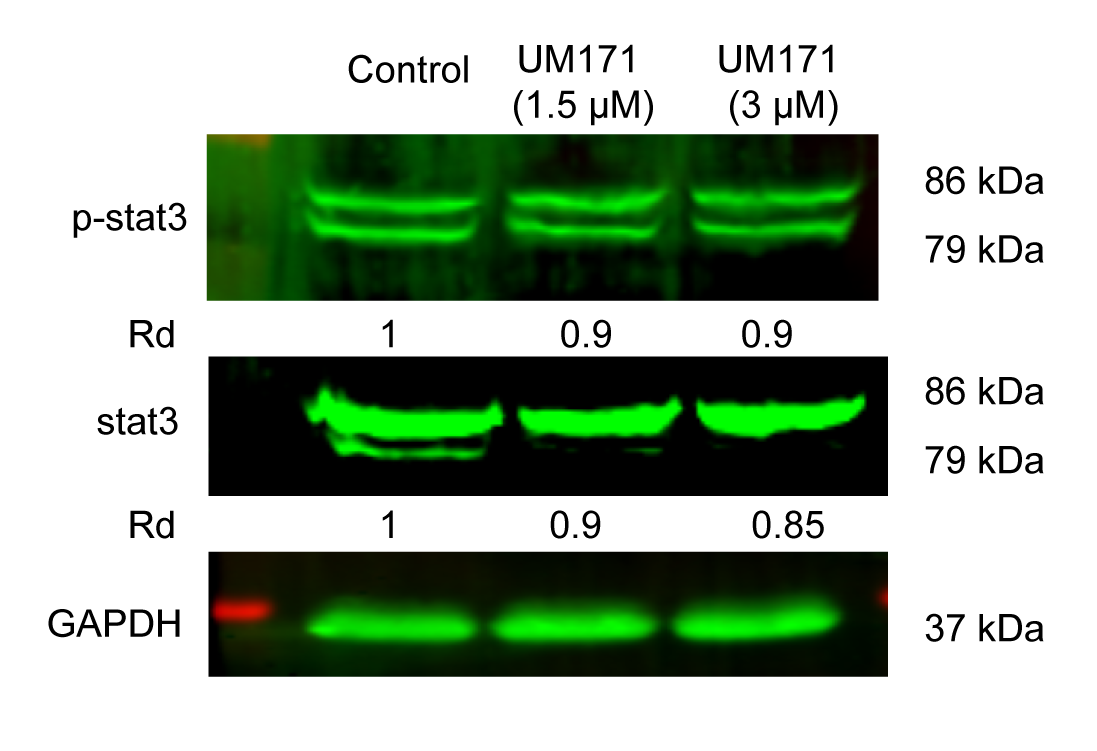

Supplement: Supplementary file 2 — supplemental figure 2 [file 41420_2022_1244_MOESM2_ESM.tif]

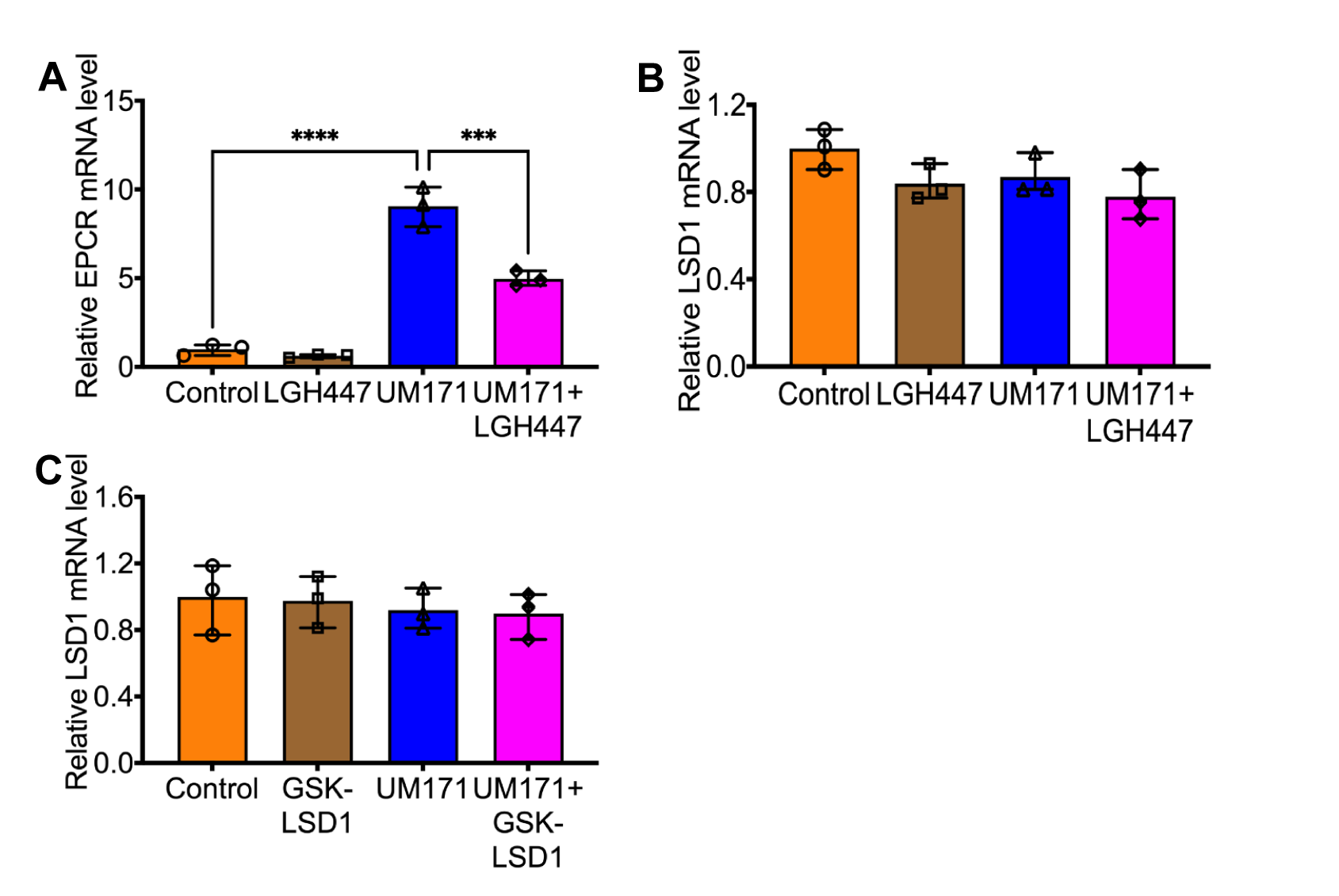

Supplement: Supplementary file 3 — supplemental figure 3 [file 41420_2022_1244_MOESM3_ESM.tif]

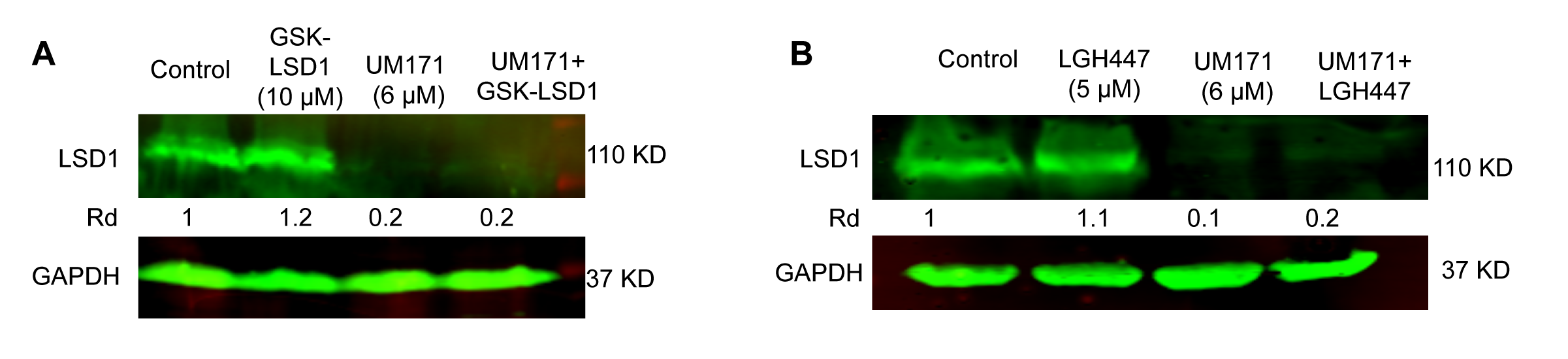

Supplement: Supplementary file 4 — supplemental figure 4 [file 41420_2022_1244_MOESM4_ESM.tif]

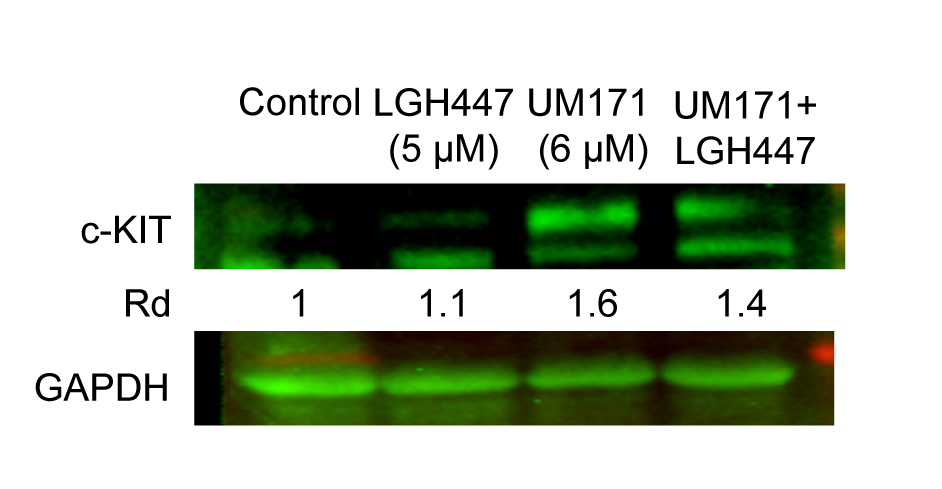

Supplement: Supplementary file 5 — supplemental figure 5 [file 41420_2022_1244_MOESM5_ESM.tif]

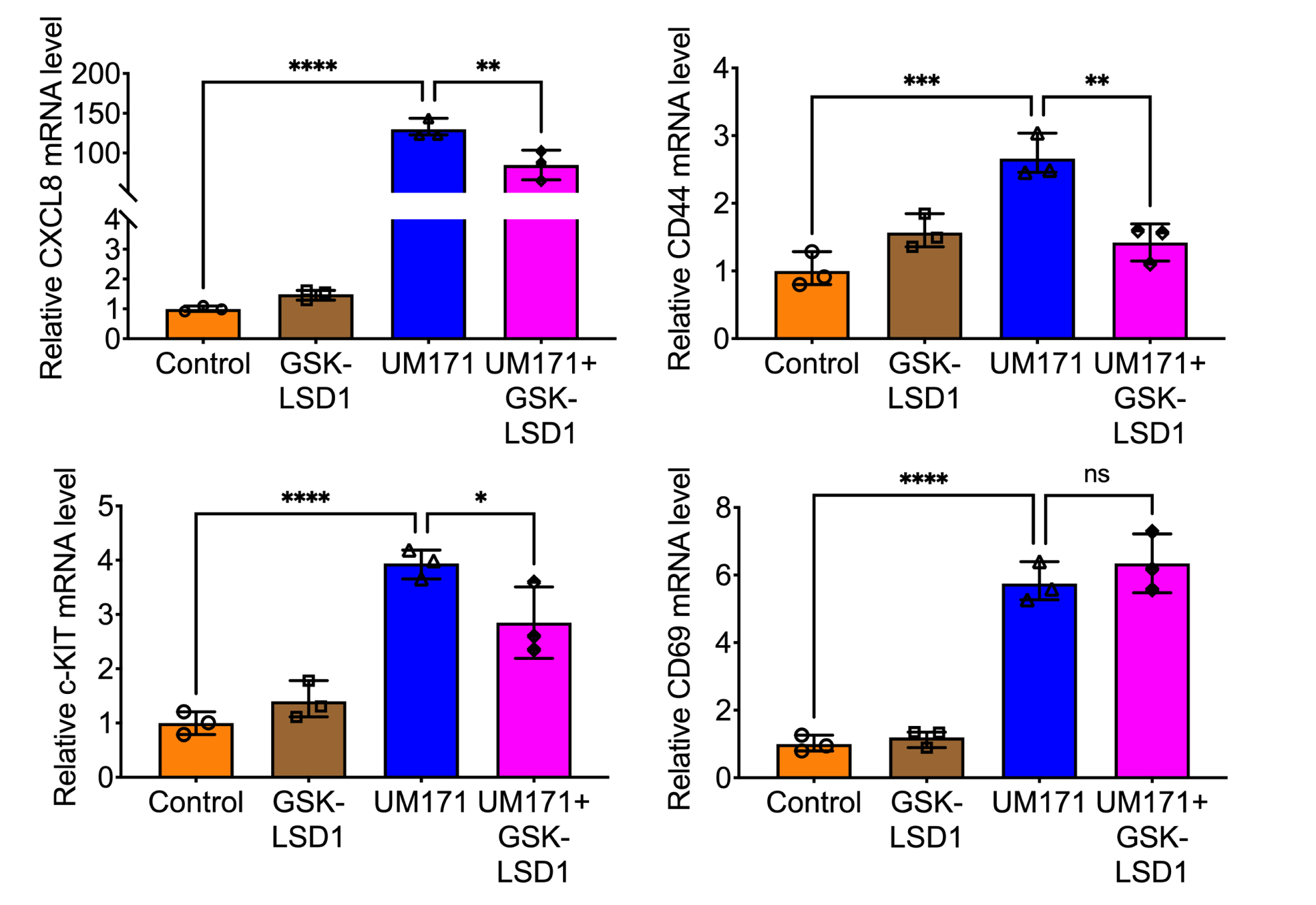

Supplement: Supplementary file 6 — supplemental figure 6 [file 41420_2022_1244_MOESM6_ESM.tif]

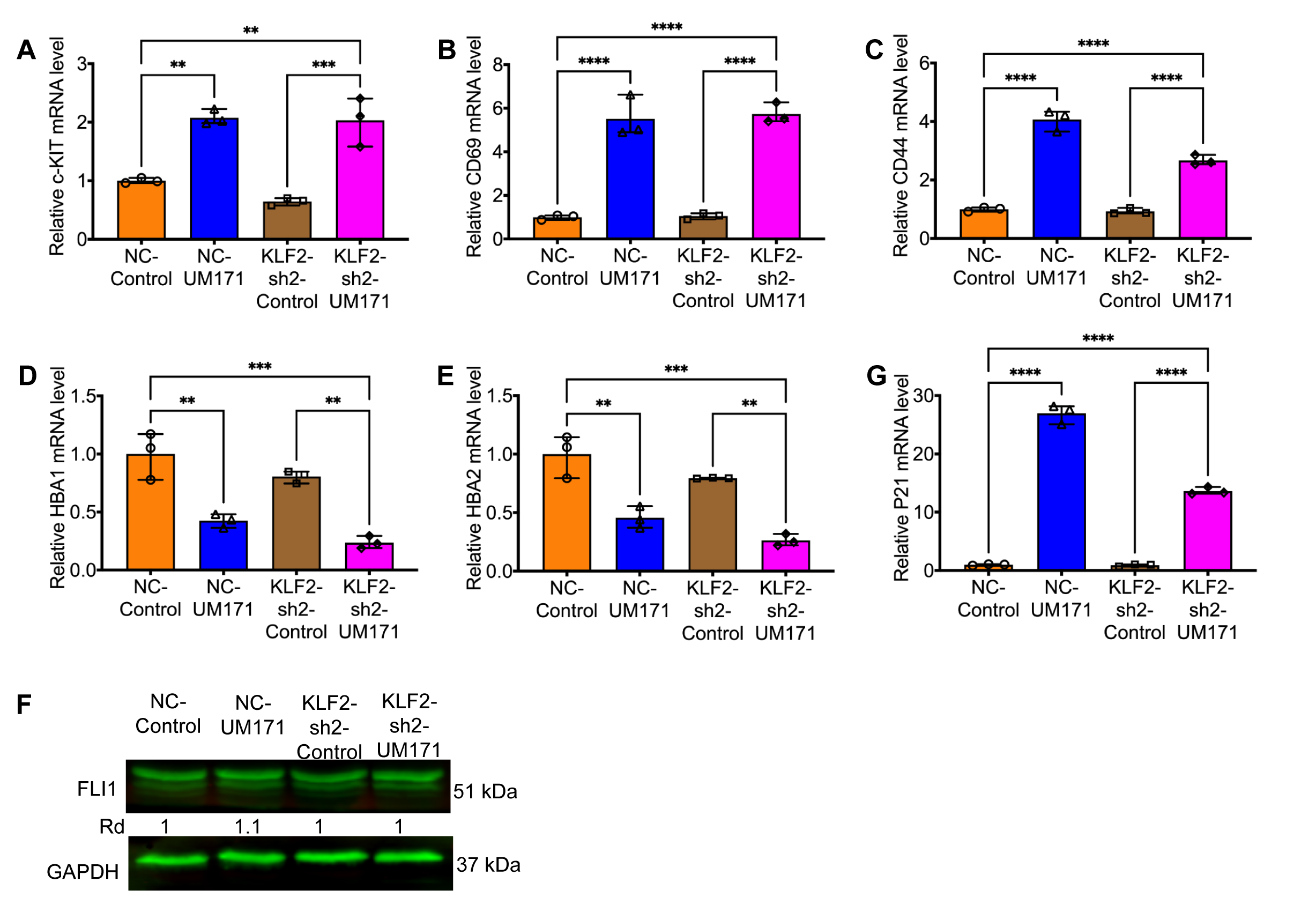

Supplement: Supplementary file 7 — supplemental figure 7 [file 41420_2022_1244_MOESM7_ESM.tif]

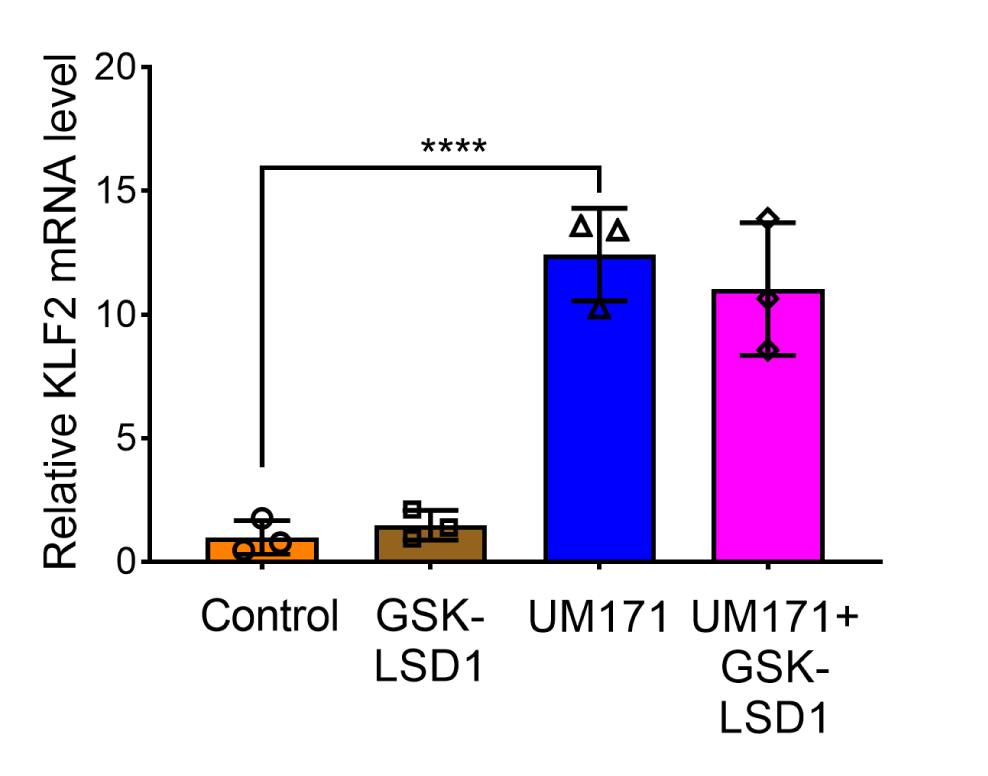

Supplement: Supplementary file 8 — supplemental figure 8 [file 41420_2022_1244_MOESM8_ESM.tif]
