# Supplementary material for: UM171 cooperates with PIM1 inhibitors to restrict HSC expansion markers and suppress leukemia progression
Source: Cell Death Discov. 2022 Nov 5;8:448. doi: 10.1038/s41420-022-01244-6 (PMC9637110; doi:10.1038/s41420-022-01244-6)
Supplement: Supplementary file 9 — SUPPLEMENTAL FIGURES LEDENDS [file 41420_2022_1244_MOESM9_ESM.docx]

**SUPPLEMENTAL FIGURES**

**Supplemental figure 1. Induction of erythroid differentiation markers by UM171 in HEL cells.** (A,B) HEL cells was treated with UM171 (3 μM) and DMSO for 24h and subjected to flow cytometry analysis for the erythroid markers CD71 (A) and CD235 (B). (C-F) Q-RT-PCR analysis of HEL cell treated with UM171 (3 μM) and DMSO for expression of the HBA1 (C), HBA2 (D), HBQ1 (D) and HBZ (F).

**Supplemental figure 2. UM171 suppresses STAT3.** HEL cells were treated with UM171 (3 μM) and DMSO for 24 hours and subjected to western blot for expression of the indicated genes.

**Supplemental figure 3. Regulation of the EPCR and LSD1 genes by UM171.** (A, C) HEL cells were treated with UM171 (6 μM), LGH447 (5 μM), UM171+LGH447 and DMSO for 24 hours and subjected to Q-RT-PCR analysis for expression of EPCR (A) and LSD1 (B). (C) HEL cells were treated with UM171 (6 μM), GSK-LSD1(10 uM), UM171+GSK-LSD1 and DMSO for 24 hours and subjected to Q-RT-PCR analysis for expression of LSD1.

**Supplemental figure 4: downregulation of LSD1 by UM171.** HEL cells were treated with the indicated drugs and subjected to western blot for expression of LSD1 (A) and GAPDH (B).

**Supplemental figure 5: Induction of c-KIT by UM171.** HEL cells were treated with the indicated drugs and subjected to western blot for expression of c-KIT and GAPDH.

**Supplemental figure 6. Induction of HSCE by UM171**. HEL cells were treated with the UM171 (6 μM) and GSK-LSD1 (10 μM) and subjected to Q-RT-PCR analysis for the indicated genes.

**Supplemental figure 7. Regulation of the HSCE genes by KLF2.** (A-E and G) KLF2 knockdown (KLF2-sh2) and control (NC) cells were treated with UM171 (6 μM), LGH447 (5 μM) and combination of two drugs for 24h and subjected to Q-RT-PCR analysis for expression of the indicated genes. (F) Expression of FLI1 in KLF2-sh2 and control cells treated with or without UM171, by western blot.

**Supplemental figure 8. LSD1 does not regulate KLF2 induction by UM171.** Q-RT-PCR expression analysis of KLF2 expression in UM171 after treatment with GSK-LSD1 (10 μM).
