# Supplementary material for: UM171 cooperates with PIM1 inhibitors to restrict HSC expansion markers and suppress leukemia progression
Source: Cell Death Discov. 2022 Nov 5;8:448. doi: 10.1038/s41420-022-01244-6 (PMC9637110; doi:10.1038/s41420-022-01244-6)
Supplement: Supplementary file 10 — Supplementary Table 1 [file 41420_2022_1244_MOESM10_ESM.docx]

Supplementary table 1 | ShRNA Sequence

|  | KLF2 shRNA |
| --- | --- |
| shRNA1 | CGGCACCGACGACGACCTCAATTCAAGAGATTGAGGTCGTCGTCGGTGCCGTTTTTT |
| shRNA2 | AGTTCGCATCTGAAGGCGCATTTCAAGAGAATGCGCCTTCAGATGCGAACTTTTTTT |
| shRNA3 | CACCGGCCATTCCAGTGCCATTTCAAGAGAATGGCACTGGAATGGCCGGTGTTTTTT |
