# Supplementary material for: UM171 cooperates with PIM1 inhibitors to restrict HSC expansion markers and suppress leukemia progression
Source: Cell Death Discov. 2022 Nov 5;8:448. doi: 10.1038/s41420-022-01244-6 (PMC9637110; doi:10.1038/s41420-022-01244-6)
Supplement: Supplementary file 11 — Supplementary Table 2 [file 41420_2022_1244_MOESM11_ESM.docx]

Supplementary table 2 | Primers for real time PCR

| Gene |  | Sequence |
| --- | --- | --- |
| CD34 | Forward | CTACAACACCTAGTACCCTTGGA |
|  | Reverse | GGTGAACACTGTGCTGATTACA |
| CD40 | Forward | GCAGGCACAAACAAGACTGA |
|  | Reverse | TCGTCGGGAAATT GATCTC |
| CD41 | Forward | TGCTGGTGTTCCTGGGTC |
|  | Reverse | AGGTCTGGGTATCCGTTGTC |
| CD44 | Forward | CTGCCGCTTTGCAGGTGTA |
|  | Reverse | CATTGTGGGCAAGGTGCTATT |
| CD69 | Forward | AGTTCCTGTCCTGTGTGCTG |
|  | Reverse | TTTGGGCTGAAGTCCAGCTC |
| C-KIT | Forward | GGGGGATCCGATGTGGGCAAGACTTCT |
|  | Reverse | CAGCAAAGGAGTGAACAG |
| CXCL8 | Forward | TTTTGCCAAGGAGTGCTAAAGA |
|  | Reverse | AACCCTCTGCACCCAGTTTTC |
| EPCR | Forward | CCTACAACCGCACTCGGTATG |
|  | Reverse | CGCGGAAATATGTTTCTGCACA |
| ERG | Forward | GGAAGCCTTATCAGTTGTGA |
|  | Reverse | GGCGGGAAGATGGTGGGCAG |
| GFI1 | Forward | CCGCGCTCATTTCTCGTCA |
|  | Reverse | ACGGAGGGAATAGTCTGGTCC |
| HBA1 | Forward | CCGGTCAACTTCAAGCTCCT |
|  | Reverse | GCCGCCCACTCAGACTTTAT |
| HBA2 | Forward | TCTCCTGCCGACAAGACCAA |
|  | Reverse | GCAGTGGC TTAGCTTGAAGTTG |
| HBQ1 | Forward | GGCGTCTACACGACAGAGG |
|  | Reverse | GCCGAGATAACGTGGCTCAG |
| HBZ | Forward | CCCGCAGACCAAGACCTAC |
|  | Reverse | ACGACCGATAGGAACTTGTCC |
| KLF2 | Forward | TTCGGTCTCTTCGACGACG |
|  | Reverse | TGCGAACTCTTGGTGTAGGTC |
| LSD1 | Forward | TGACCGGATGACTTCTCAAGA |
|  | Reverse | GTTGGAGAGTAGCCTCAAATGTC |
| P21 | Forward | TGTCCGTCAGAACCCATGC |
|  | Reverse | AAAGTCGAAGTTCCATCGCTC |
| PIM1 | Forward | GGCTCGGTCTACTCAGGCA |
|  | Reverse | GGAAATCCGGTCCTTCTCCAC |
| PIM2 | Forward | TTGACCAAGCCTCTACAGGG |
|  | Reverse | CCACCTGGAGTCGATCTGTGA |
| PIM3 | Forward | AAGGACGAAAATCTGCTTGTGG |
|  | Reverse | CGAAGTCGGTGTAGACCGTG |
| ZFP36L1 | Forward | ACTCCAGCCGCTACAAGAC |
|  | Reverse | CGTAGGGGCAAAAGCCGAT |
