# Supplementary material for: UM171 cooperates with PIM1 inhibitors to restrict HSC expansion markers and suppress leukemia progression
Source: Cell Death Discov. 2022 Nov 5;8:448. doi: 10.1038/s41420-022-01244-6 (PMC9637110; doi:10.1038/s41420-022-01244-6)
Supplement: Supplementary file 13 — full length of western blot [file 41420_2022_1244_MOESM13_ESM.pdf]

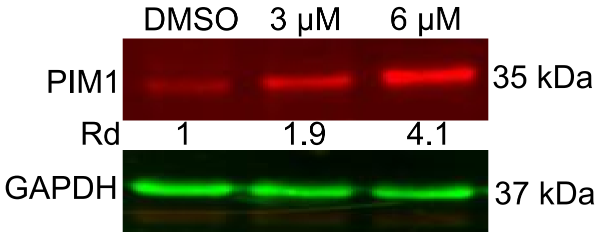

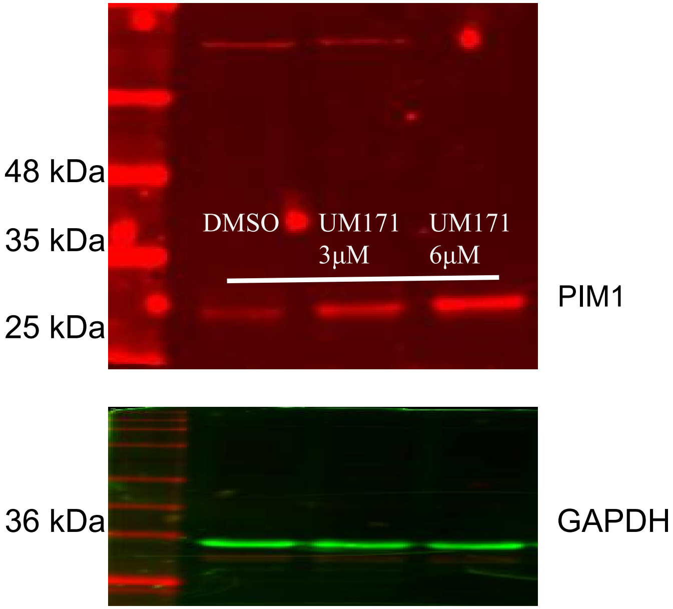

PIM1-pull down

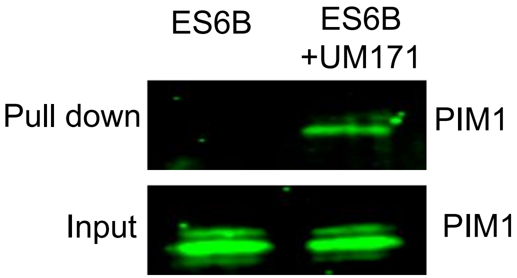

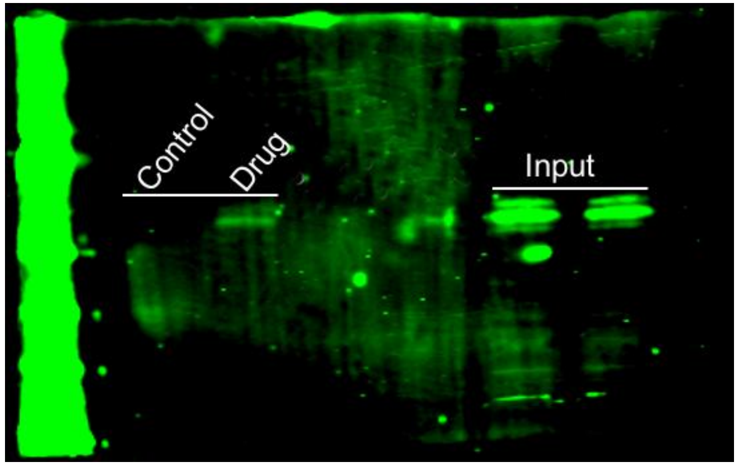

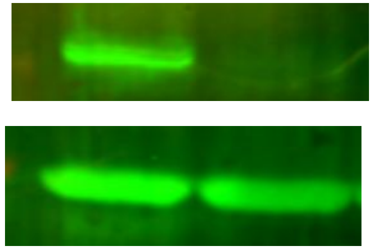
Control
UM171
LSD1
110 KD
GAPDH
37 KD

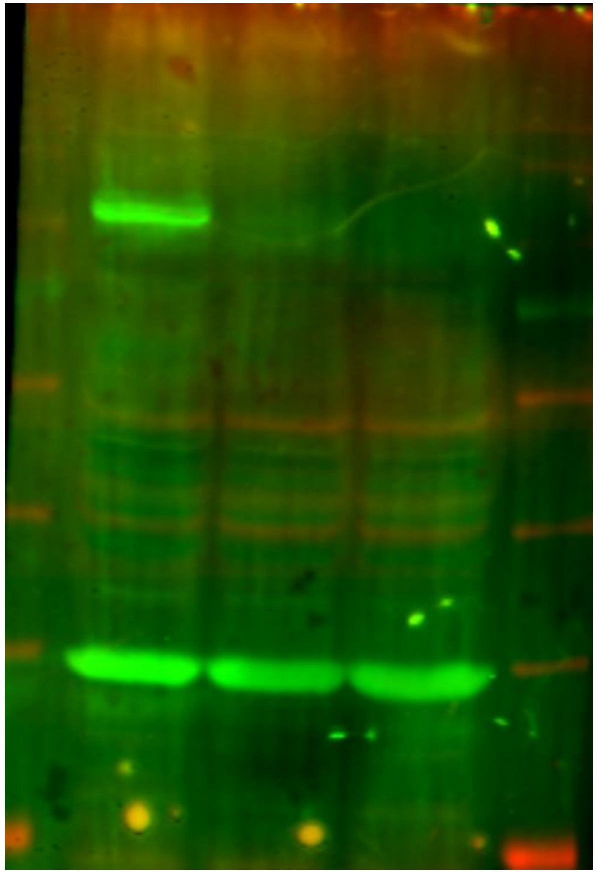
Control
UM171
110 KD
LSD1
37 KD
GAPDH

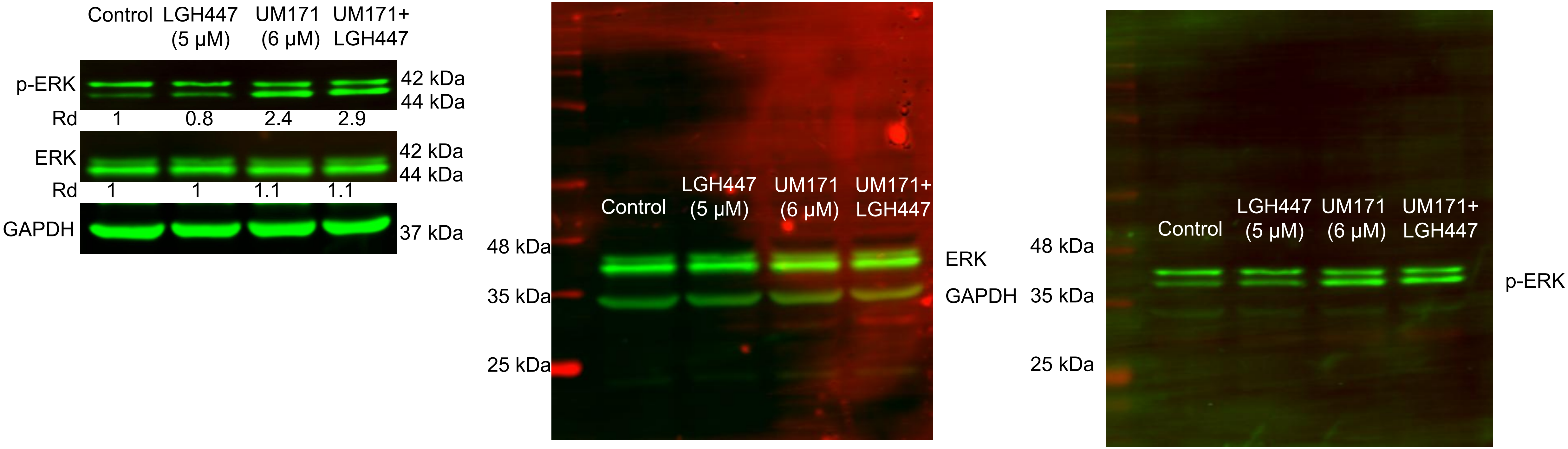

Control
LGH447 (5 μM)
UM171 (6 μM)
UM171+ LGH447
p-ERK
42 kDa
44 kDa
Rd 1 0.8 2.4 2.9
ERK
42 kDa
44 kDa
Rd 1 1 1.1 1.1
GAPDH
37 kDa
Control
LGH447 (5 μM)
UM171 (6 μM)
UM171+ LGH447
48 kDa
35 kDa
25 kDa
ERK
GAPDH
Control
LGH447 (5 μM)
UM171 (6 μM)
UM171+ LGH447
48 kDa
35 kDa
25 kDa
p-ERK

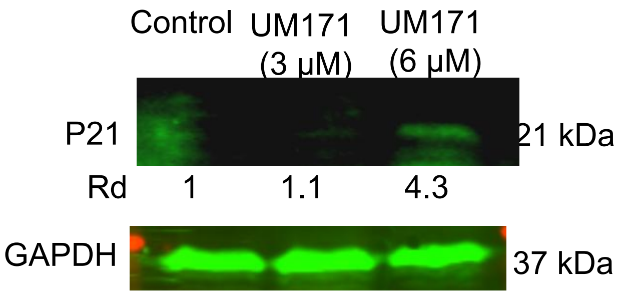
Control
UM171 (3 μM)
UM171 (6 μM)
P21
21 kDa
Rd
1
1.1
4.3
GAPDH
37 kDa

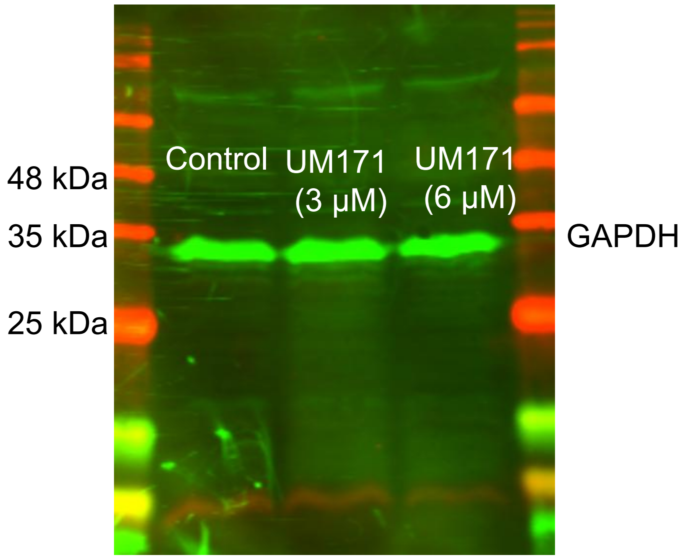
48 kDa
35 kDa
25 kDa
Control
UM171 (3 μM)
UM171 (6 μM)
GAPDH

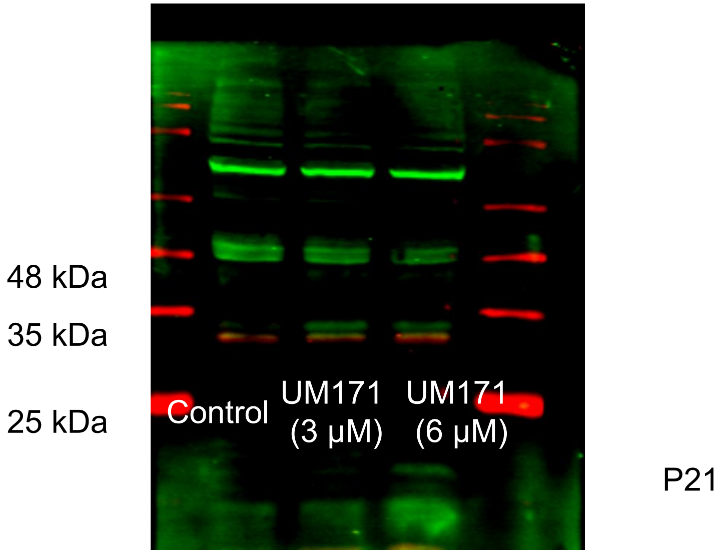
48 kDa
35 kDa
25 kDa
Control
UM171 (3 μM)
UM171 (6 μM)
P21

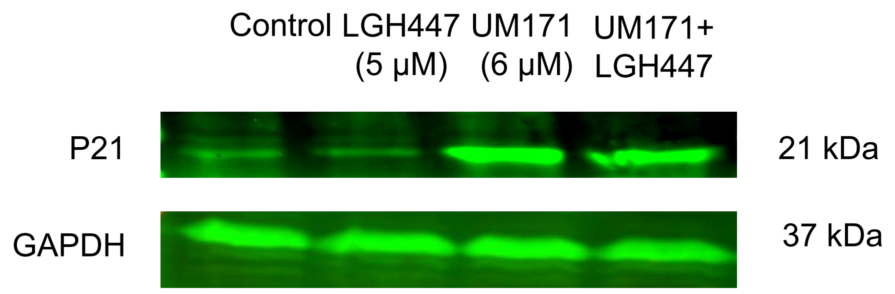

Control
LGH447 (5 μM)
UM171 (6 μM)
UM171+ LGH447
P21
21 kDa
GAPDH
37 kDa

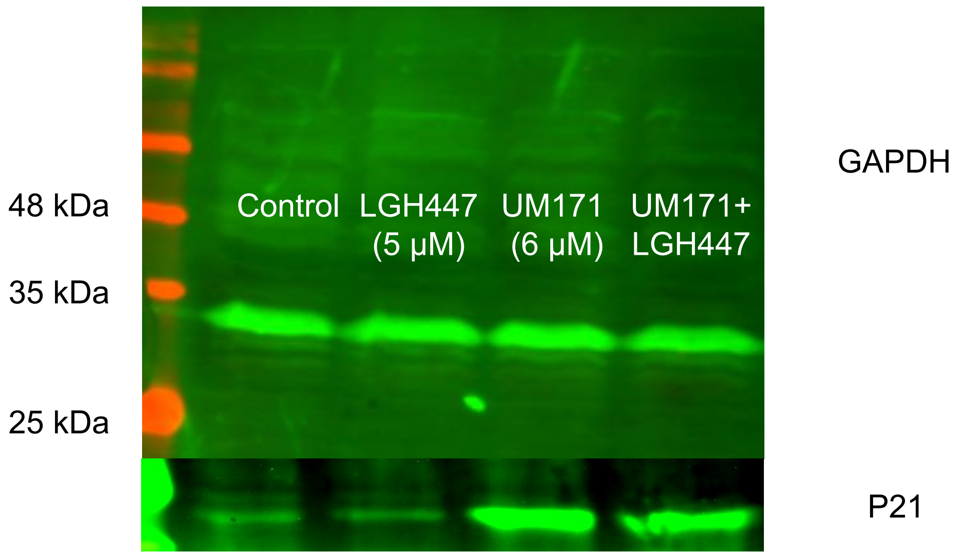

48 kDa
35 kDa
25 kDa
Control
LGH447 (5 μM)
UM171 (6 μM)
UM171+ LGH447
GAPDH
P21

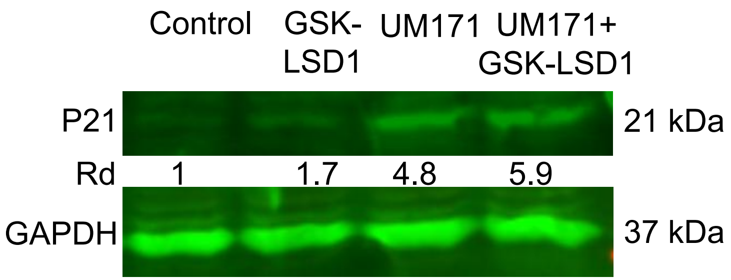
Control
GSK-LSD1
UM171
UM171+ GSK-LSD1
P21
21 kDa
Rd
1
1.7
4.8
5.9
GAPDH
37 kDa

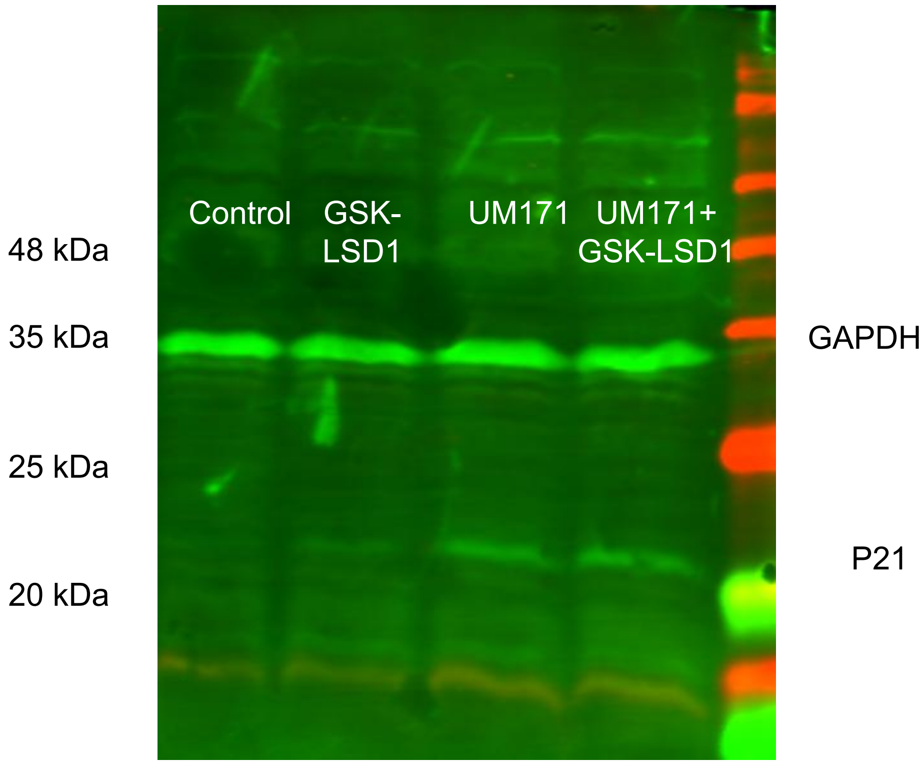
Control
GSK-LSD1
UM171
UM171+ GSK-LSD1
48 kDa
35 kDa
25 kDa
20 kDa
GAPDH
P21

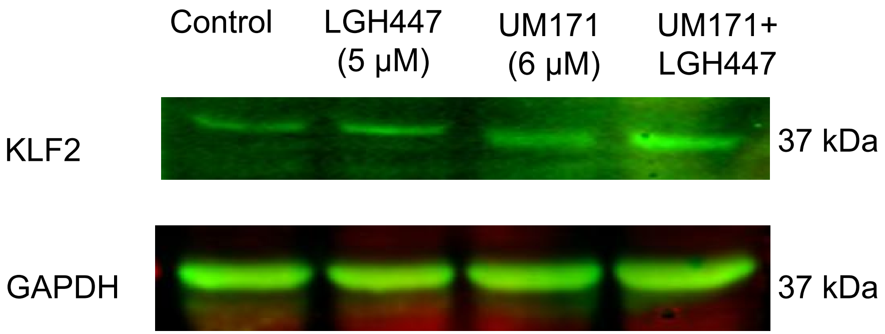
Control
LGH447 (5 μM)
UM171 (6 μM)
UM171+ LGH447
KLF2
37 kDa
GAPDH
37 kDa

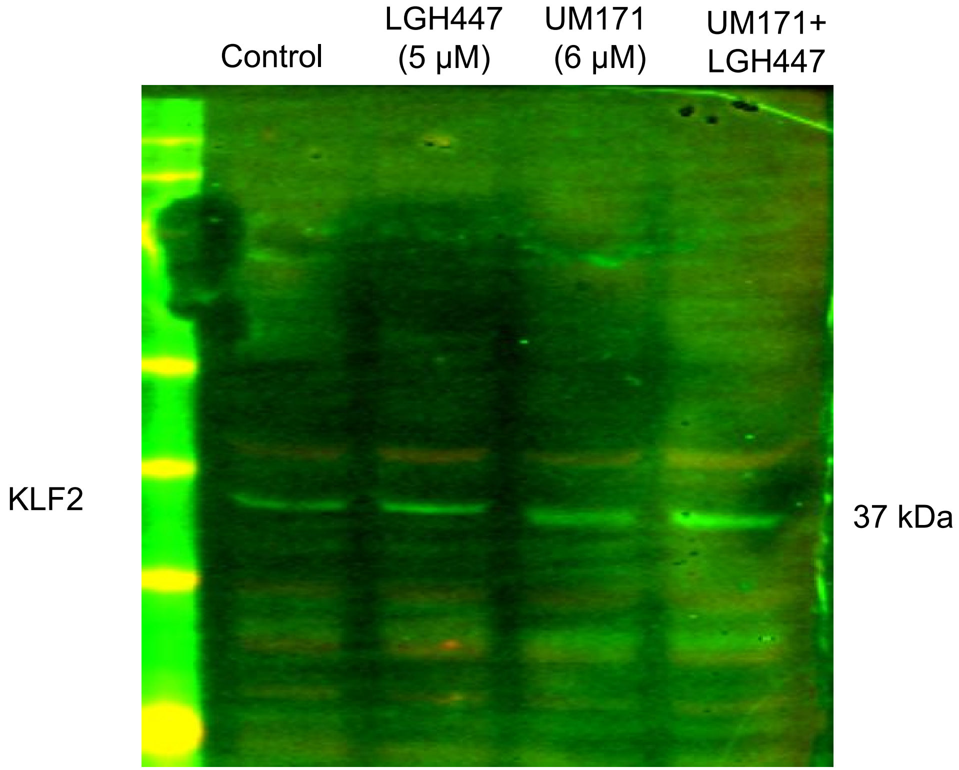
Control
LGH447 (5 μM)
UM171 (6 μM)
UM171+ LGH447
KLF2
37 kDa

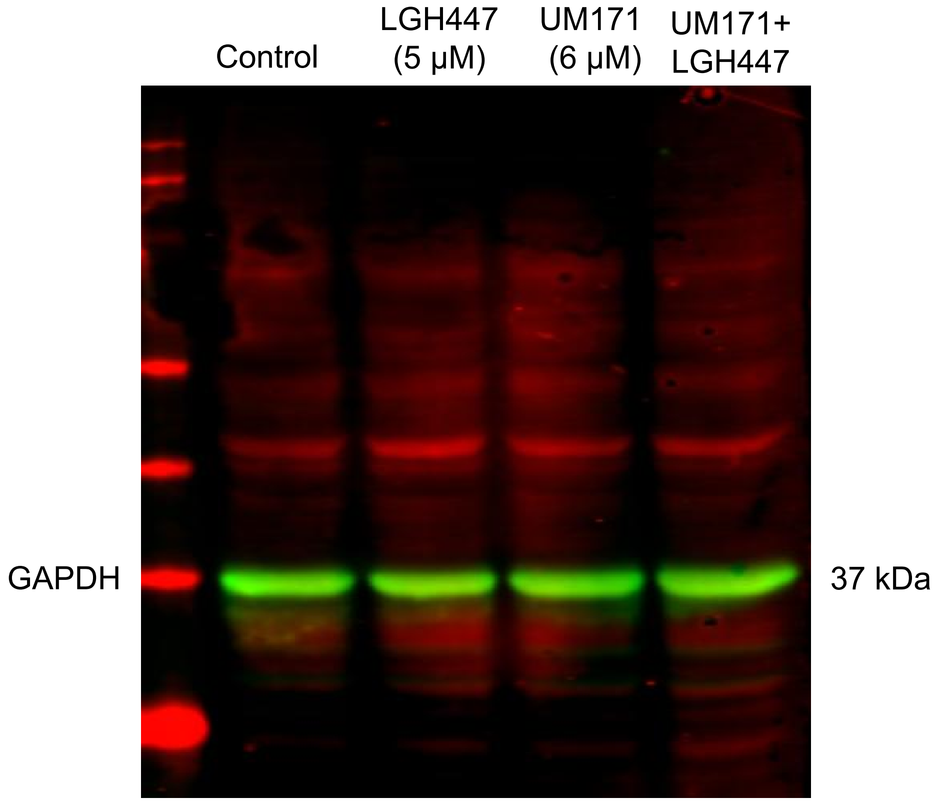
Control
LGH447 (5 μM)
UM171 (6 μM)
UM171+ LGH447
GAPDH
37 kDa

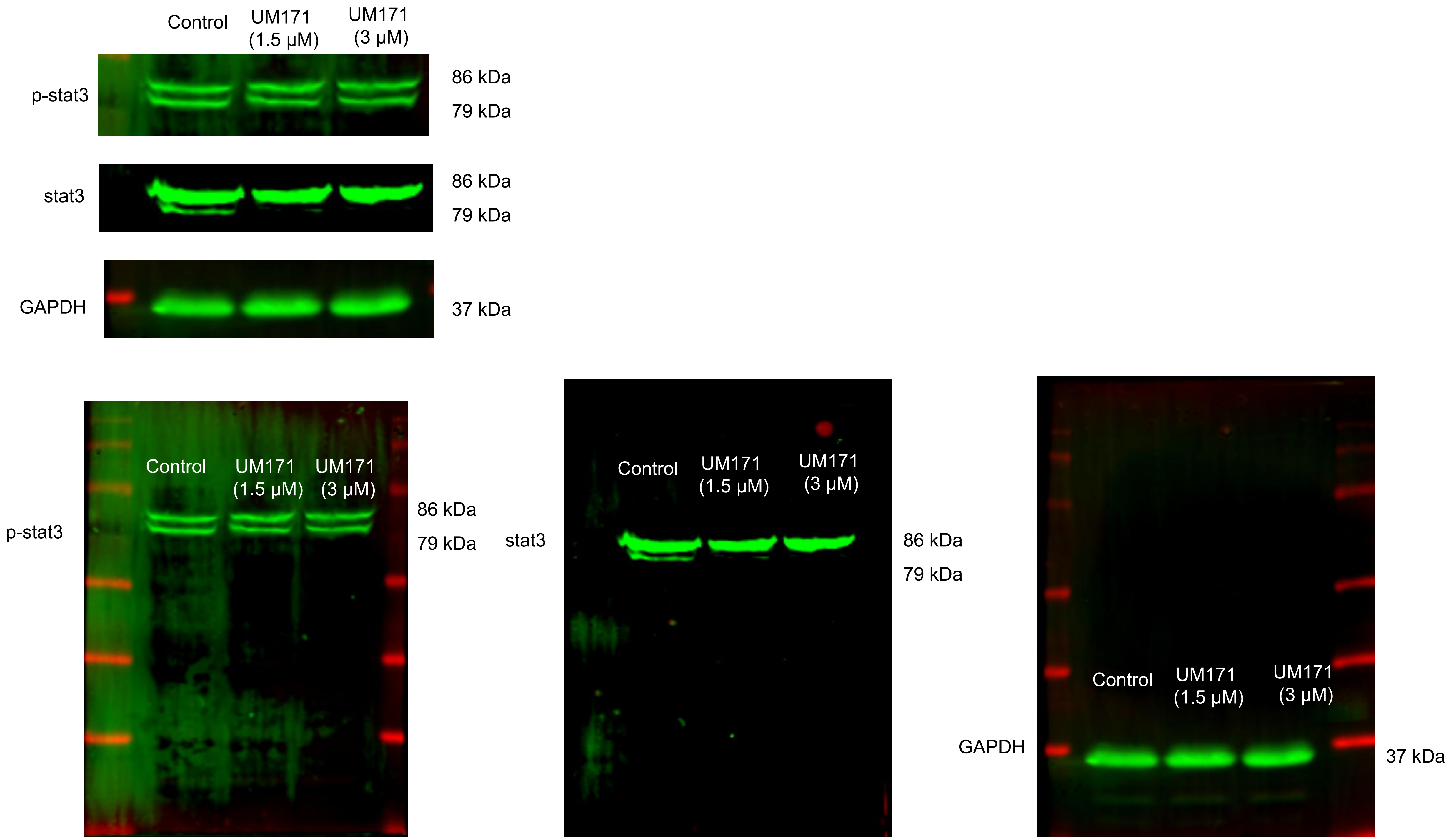
Control
UM171 (1.5 μM)
UM171 (3 μM)
p-stat3
86 kDa
79 kDa
stat3
86 kDa
79 kDa
GAPDH
37 kDa
p-stat3
Control
UM171 (1.5 μM)
UM171 (3 μM)
86 kDa
79 kDa
stat3
Control
UM171 (1.5 μM)
UM171 (3 μM)
86 kDa
79 kDa
GAPDH
Control
UM171 (1.5 μM)
UM171 (3 μM)
37 kDa

Control | GSK-LSD1 (10 µM) | UM171 (6 µM) | UM171+ GSK-LSD1

LSD1 110 KD

GAPDH 37 KD

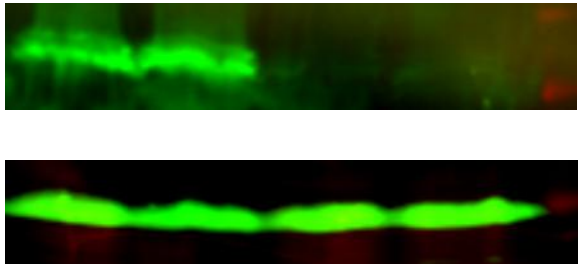

Control | GSK-LSD1 (10 µM) | UM171 (6 µM) | UM171+ GSK-LSD1

LSD1 110 KD

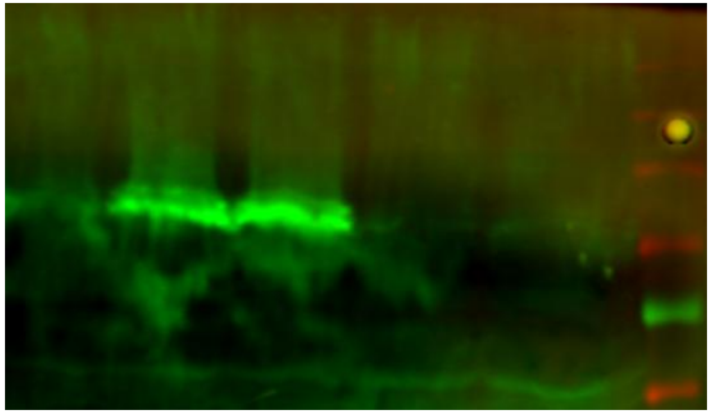

GAPDH 37 KD

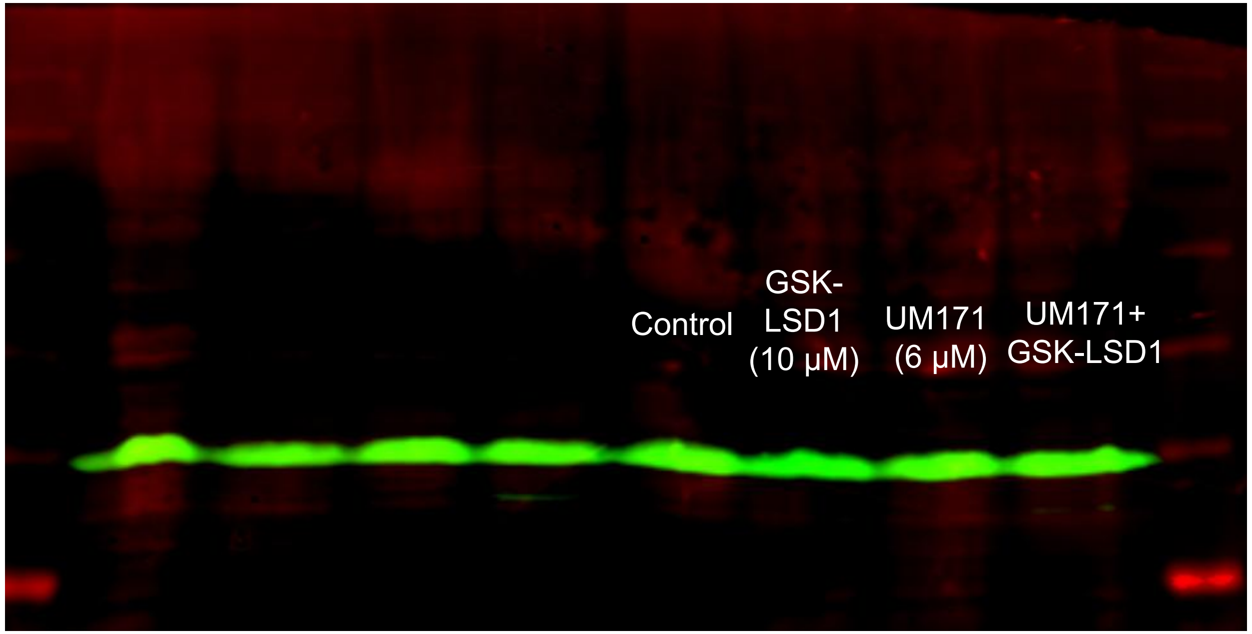

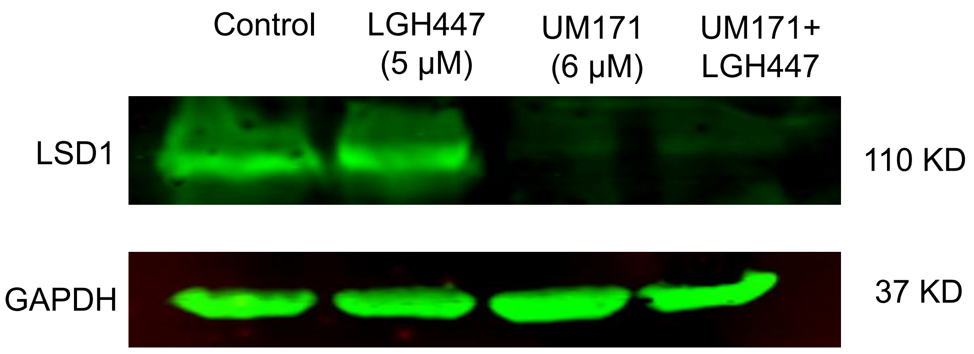
Control
LGH447 (5 μM)
UM171 (6 μM)
UM171+ LGH447
LSD1
110 KD
GAPDH
37 KD

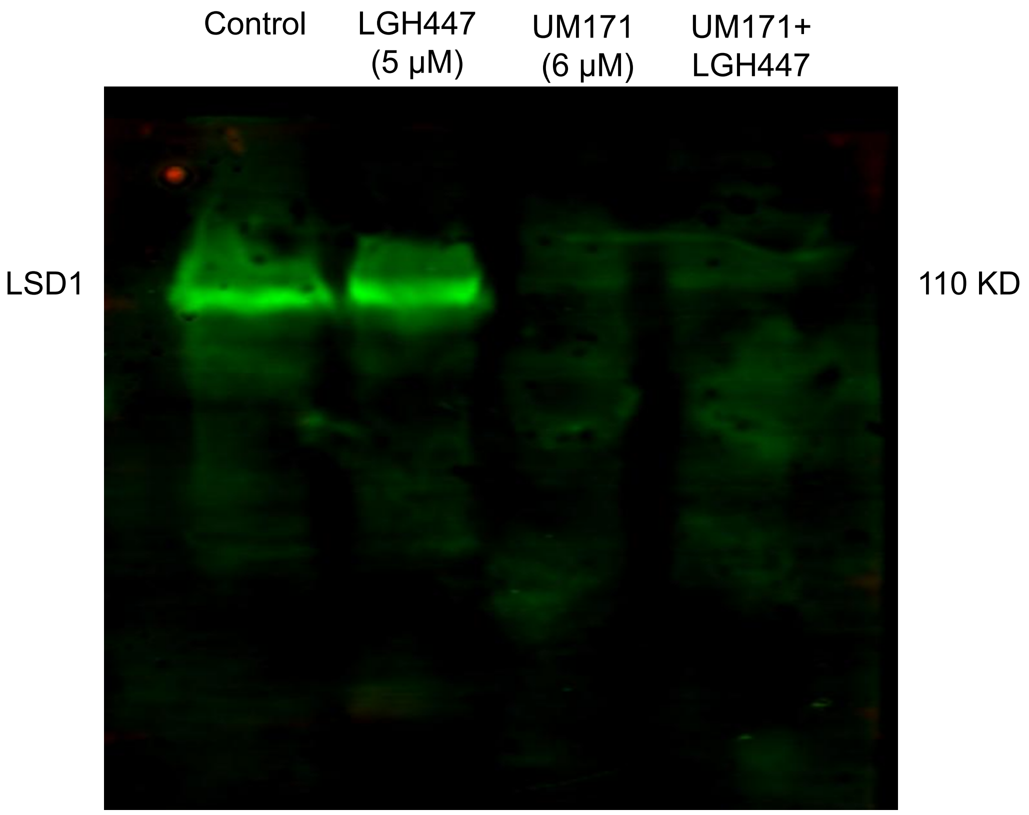
Control
LGH447 (5 μM)
UM171 (6 μM)
UM171+ LGH447
LSD1
110 KD

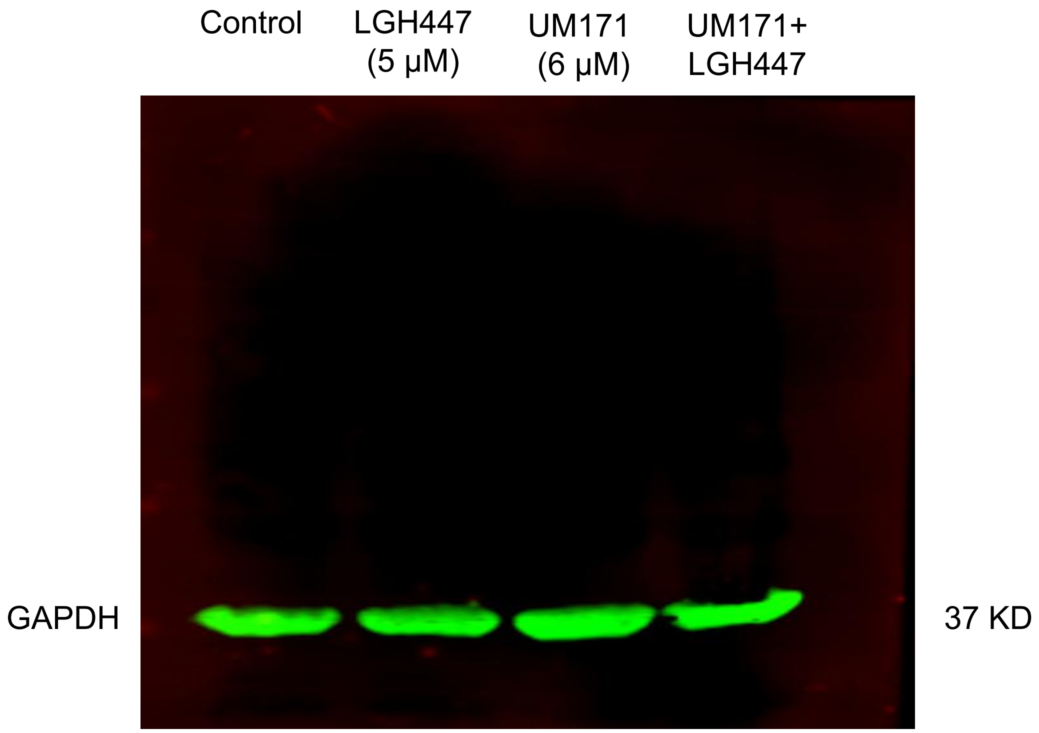
Control
LGH447 (5 μM)
UM171 (6 μM)
UM171+ LGH447
GAPDH
37 KD

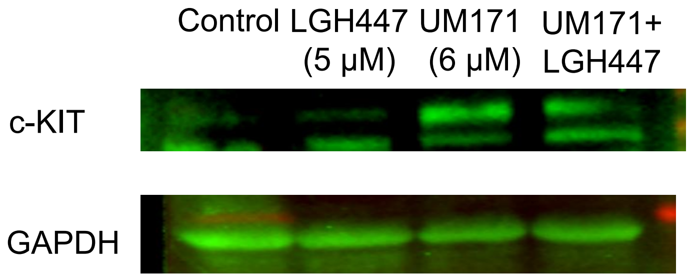
Control
LGH447 (5 μM)
UM171 (6 μM)
UM171+ LGH447
c-KIT
GAPDH

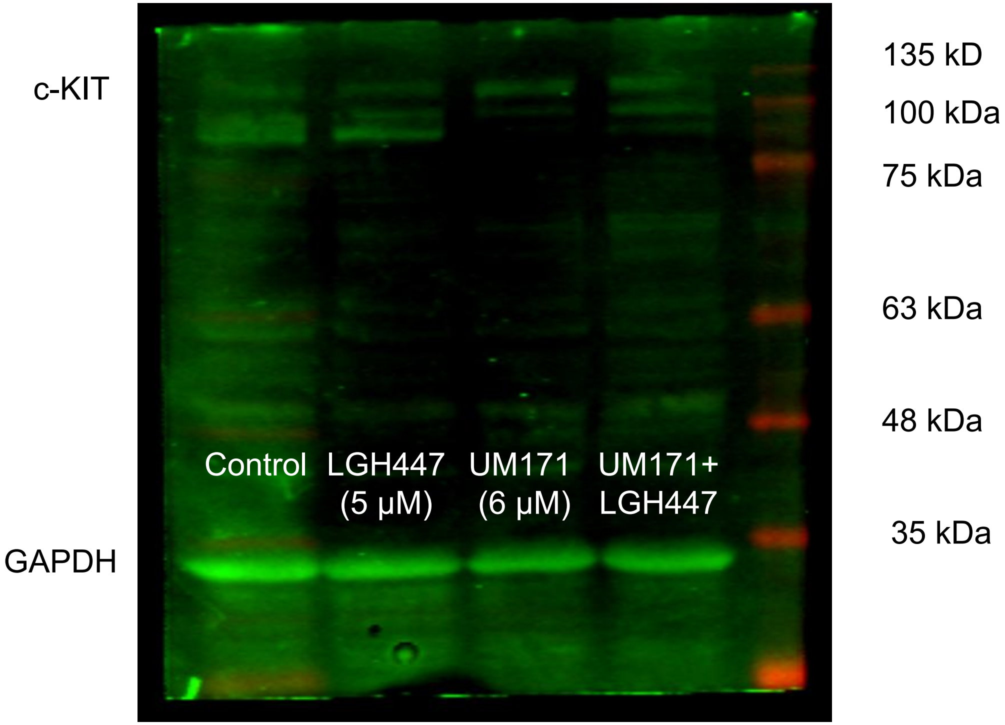
c-KIT
GAPDH
Control
LGH447 (5 μM)
UM171 (6 μM)
UM171+ LGH447
135 kD
100 kDa
75 kDa
63 kDa
48 kDa
35 kDa

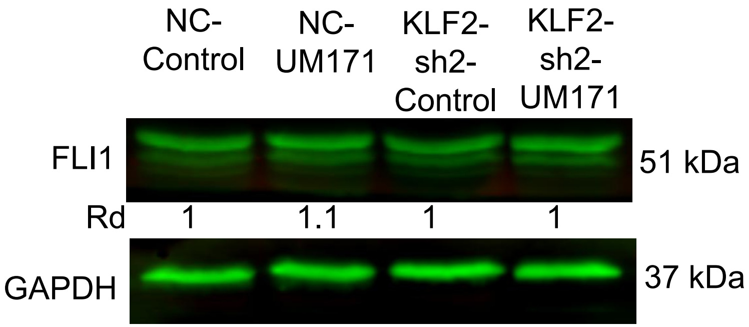
NC-Control
NC-UM171
KLF2-sh2-Control
KLF2-sh2-UM171
FLI1
51 kDa
Rd
1
1.1
1
1
GAPDH
37 kDa

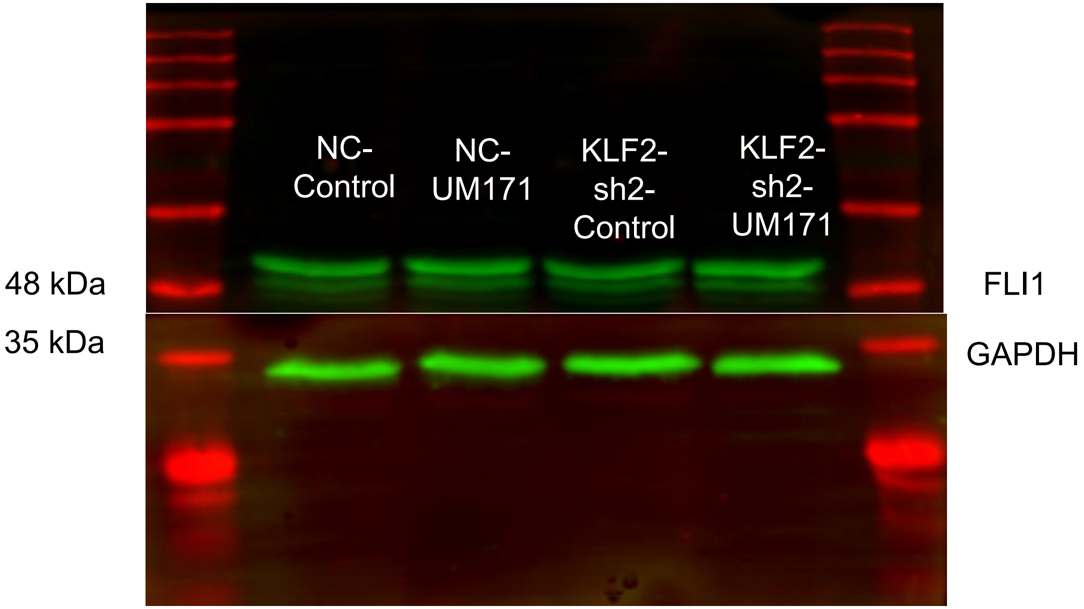
NC-Control
NC-UM171
KLF2-sh2-Control
KLF2-sh2-UM171
48 kDa
FLI1
35 kDa
GAPDH
